# Machine learning and the prediction of suicide in psychiatric populations: a systematic review

**DOI:** 10.1038/s41398-024-02852-9

**Published:** 2024-03-09

**Authors:** Alessandro Pigoni, Giuseppe Delvecchio, Nunzio Turtulici, Domenico Madonna, Pietro Pietrini, Luca Cecchetti, Paolo Brambilla

**Affiliations:** 1 Social and Affective Neuroscience Group, MoMiLab, IMT School for Advanced Studies Lucca, Lucca, Italy; 2 Department of Neurosciences and Mental Health, Fondazione IRCCS Ca’ Granda, Ospedale Maggiore Policlinico, Milan, Italy; 3 Department of Pathophysiology and Transplantation, University of Milan, Milan, Italy; 4 MoMiLab, IMT School for Advanced Studies Lucca, Lucca, Italy

**Keywords:** Bipolar disorder, Prognostic markers

## Abstract

Machine learning (ML) has emerged as a promising tool to enhance suicidal prediction. However, as many large-sample studies mixed psychiatric and non-psychiatric populations, a formal psychiatric diagnosis emerged as a strong predictor of suicidal risk, overshadowing more subtle risk factors specific to distinct populations. To overcome this limitation, we conducted a systematic review of ML studies evaluating suicidal behaviors exclusively in psychiatric clinical populations. A systematic literature search was performed from inception through November 17, 2022 on PubMed, EMBASE, and Scopus following the PRISMA guidelines. Original research using ML techniques to assess the risk of suicide or predict suicide attempts in the psychiatric population were included. An assessment for bias risk was performed using the transparent reporting of a multivariable prediction model for individual prognosis or diagnosis (TRIPOD) guidelines. About 1032 studies were retrieved, and 81 satisfied the inclusion criteria and were included for qualitative synthesis. Clinical and demographic features were the most frequently employed and random forest, support vector machine, and convolutional neural network performed better in terms of accuracy than other algorithms when directly compared. Despite heterogeneity in procedures, most studies reported an accuracy of 70% or greater based on features such as previous attempts, severity of the disorder, and pharmacological treatments. Although the evidence reported is promising, ML algorithms for suicidal prediction still present limitations, including the lack of neurobiological and imaging data and the lack of external validation samples. Overcoming these issues may lead to the development of models to adopt in clinical practice. Further research is warranted to boost a field that holds the potential to critically impact suicide mortality.

*“Is there no way out of the mind?”*

-Sylvia Plath

“*The person in whom Its invisible agony reaches a certain unendurable level will kill herself the same way a trapped person will eventually jump from the window of a burning high-rise. Make no mistake about people who leap from burning windows. Their terror of falling from a great height is still just as great as it would be for you or me standing speculatively at the same window just checking out the view; i.e., the fear of falling remains a constant. The variable here is the other terror, the fire’s flames: when the flames get close enough, falling to death becomes the slightly less terrible of two terrors*.

*It’s not desiring the fall; it’s terror of the flames.”*

- David Foster Wallace

## Introduction

The prediction of suicide has been a challenge for decades, and to date, a method for anticipating individual suicides or stratifying patients according to suicide risk is still lacking [1]. Suicide is a worldwide phenomenon and ranks as the second most frequent cause of premature mortality in individuals between 15 and 29 years (preceded only by traffic accidents), and as the third in the age group 15–44 years [2].

Alarmingly, recent studies suggest that the detection of risk factors and the implementation of interventions are inadequate [3]. The majority of individuals who have attempted suicide are reported to consult with physicians prior to the attempt, suggesting that a possibility to intervene might be possible in these help-seeking subjects. The difficulty in predicting suicidal behaviors relies on the lack of clear psychiatric biomarkers and the poor predictive power of individual risk factors [4]. Suicidal behaviors, as many other psychiatric phenomena, are likely the result of the complex relationship between several environmental and trait variables interacting to modify the actual risk rate [4, 5]. Well-recognized risk factors for suicide encompass mental disorders, previous suicide attempts, early trauma, negative life events, and vulnerable periods, with important differences among sexes in terms of ideation and lethality [6, 7]. However, traditional suicide risk factors have only limited clinical predictive value and show a relatively poor clinical utility in predicting suicide occurrence [8, 9], even in high-risk population, such as depressed patients [10].

That is, to date, a method for anticipating suicides or stratifying patients according to risk for suicidal behaviors remains elusive, and no biomarkers have been yet established [9, 11].

Over the last decades, machine learning (ML) techniques emerged as a potential new tool to improve the management of complex problems in psychiatry [12]. This form of multimodal learning has shown to improve prognostic/predictive performance in various fields of medicine, e.g., cardiology and neurology [13, 14]. As a matter of fact, ML can be used to process high-dimensional sets of variables and determine the optimal model for classification. Importantly, such techniques allow predictions at the individual level, therefore representing a promising tool to accurately characterize the complex nature of suicidal behavior.

In the last few years, several algorithms and procedures have been used to predict suicidal behaviors in different populations [11, 15–17]. Given that suicide is considered a transdiagnostic feature, a number of studies have been conducted in the general population, sometimes with very large and heterogeneous samples [6, 18]. One of the most solid findings emerging from studies focusing on the general population is that a formal psychiatric diagnosis is a strong predictor of suicidal risk in different samples across countries [1, 6, 18, 19]. This is not surprising, as up to 90% of all suicides occur in psychiatric populations [1, 20–22], with mood disorders being considered the leading cause of suicidality among mental disorders [23, 24].

Therefore, the inclusion of both healthy individuals and psychiatric patients into large sample ML studies may prevent the identification of more subtle risk factors specific to distinct psychiatric disorders by merely taking into account a previous psychiatric diagnosis as the driving factor for the analysis. Instead, by targeting vulnerable populations only, ML could uncover predictors of suicidal behaviors specific to distinct disorders and help in better stratifying patients according to the actual risk. This would translate into useful information that can be more easily applied in clinical and forensic settings [25].

In this context, in this work, we provide a systematic review of the results from ML studies in psychiatric clinical populations and discuss crucial issues in ML literature, including employed algorithms, features, and samples, with the aim of providing meaningful considerations to future research in the field of suicide prevention.

## Material and method

The current systematic review followed the preferred reporting items for systematic reviews and meta-analyses (PRISMA) guidelines [26].

### Search strategy

A systematic literature search was performed for articles published from inception through November 17, 2022 on PubMed, EMBASE, and Scopus, using the following search terms adapted for each database:

(suicid* AND (machine learning OR support vector machine OR deep learning OR neural network OR random forest OR xboost OR gradient boosting OR regression tree OR elastic net) AND (psychiatr* OR schizophren* OR depress* OR obsessive OR bipolar OR mania OR manic OR anxiety OR borderline OR personality)

**Fig. 1:**
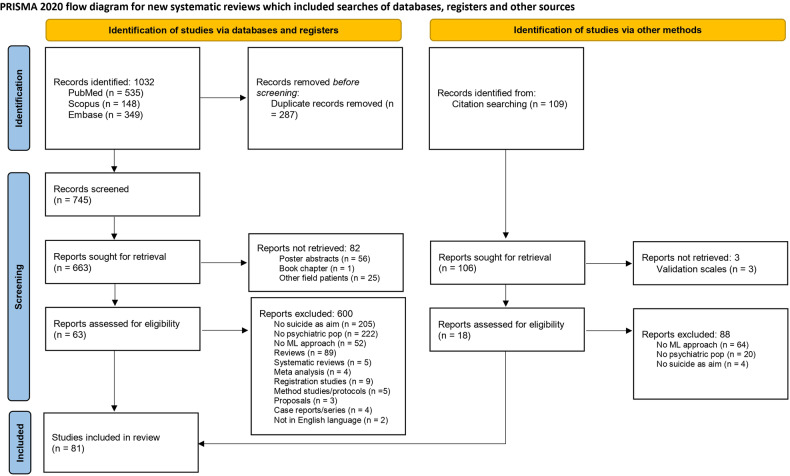
PRISMA flowchart of the study selection. Flowchart summary of the study selection process (adapted from PRISMA guidelines; Page et al., 2021).

Database searches were supplemented by hand-search, which encompassed an extensive search through the reference list of included papers, previous reviews, and the “Similar Articles” sections in PubMed (reported in Fig. 1 as “Other sources”).

Two authors (A.P. and G.D.) independently performed the literature search. Documents were assessed according to the following inclusion criteria: (1) journal article available in English, (2) original investigation, (3) employment of ML methodology, (4) evaluation of a suicide risk outcome or self-harm; (5) evaluation of a psychiatric population. Also, we included studies if (a) the sample was composed of individuals with a confirmed psychiatric diagnosis, irrespective of the specific diagnosis and disease severity, and (b) used multiple psychiatric diagnoses or a transdiagnostic framework. The absence of a control group of healthy individuals was not considered an exclusion criterion. To be included, studies must have used ML as a primary or secondary analysis method to predict suicide attempt, suicide risk, or to stratify patients according to risk. No restriction of age was applied. If controversies emerged in the screening processes, they were resolved by discussion between the two authors (A.P. and G.D.) with a third party (P.B.).

Exclusion criteria were the following: (1) non-original investigations (reviews, expert opinions, meta-analyses); (2) article not in English; (3) employment of a methodology other than ML (logistic regression was excluded, except when it was compared to other ML approaches); (4) evaluation of outcomes other than suicide; (5) exclusive evaluation of non-psychiatric populations (e.g., general population, neurologic patients, high-risk populations, emergency department patients). Given that suicidal behaviors are reported across all ages, age-related variables were not considered an exclusion criterion.

We also excluded studies in which the sample was composed by “suicide attempters” without further differentiation in terms of the presence or absence of psychiatric diagnoses. A PRISMA flowchart (Fig. 1) (Page et al., 2021) was created to graphically depict the inclusion/exclusion of studies.

### Data extracted

A preliminary data extraction form was designed by A.P.; it was then pilot-tested on five randomly selected studies and fine-tuned accordingly. The search was rerun on a weekly basis, and data from the newly included studies were added to the database accordingly.

For each article, the following variables were extracted:

- General information (author, year of publication).
- Sample characteristics (demographics, numerosity, clinical data).
- Type of ML algorithm(s) employed.
- Number and characteristics of features employed for prediction.
- ML performance metrics (AUC, Accuracy, Sensitivity, Specificity).
- Number of psychiatric diagnoses assessed.
- Type of psychiatric disorders assessed.
- Findings regarding the prediction of suicide or the classification of risk.

### Descriptive analyses

Given the different types of features and algorithms employed, the data were not homogeneous enough to be included in a quantitative meta-analysis. Descriptive analyses were employed to analyze study findings by key design characteristics such as the employed features, sample size, and ML algorithms.

### Quality assessment

An assessment for bias risk was performed using the Transparent Reporting of a Multivariable Prediction Model for Individual Prognosis or Diagnosis (TRIPOD) guidelines [27] (see Supplementary Materials for more details; see Supplementary Table 3 for risk bias results).

## Results

Based on the search strings and after the removal of duplicates, 745 unique studies were retrieved and screened for eligibility from direct database search and 109 from other sources (Fig. 1).

During this screening phase, 82 studies were rejected because they failed to fully meet the inclusion criteria. Subsequently, we reviewed the full texts of the remaining 663 studies plus 109 from other sources. Six hundred studies were further excluded since they did not meet the inclusion criteria (see Fig. 1 for a complete description).

As a result, the remaining 81 studies were included in the qualitative synthesis of the review, whose information are summarized in Table 1.

**Table 1:** Sociodemographic and clinical characteristics of the reviewed studies.

Author and year	Sample (F/M)	Population	Medication	ML algorithm (CV)	Features	Suicide measure/outcome	Outcome Metrics	Findings
Wang et al., [77]	1249 patients with suicide attempts (846/403); 11,851 patients without suicide attempts (6617/5234)	Mood disorders	Not specified	LR SVM CNN	Sociodemographic features and Text mining	Suicide attempts	LR Acc: 0.97 AUC: 0.94 Precision: 0.91 Recall: 0.74 SVM Acc: 0.97 AUC: 0.94 Precision: 0.88 Recall: 0.84 CNN Acc: 0.98 AUC: 0.97 Precision: 0.94 Recall: 0.89	Females, the elderly (>59 years old), students, the divorced or widowed, and patients with smoking, psychoactive substance dependence, and a family history of suicide had a higher risk of attempted suicide. CNN resulted the most accurate prediction model.
Chen et al., [55]	36 MDD with low-risk (19/17); 126 MDD with high-risk (86/40)	MDD	Unmedicated	SVM (LOOCV)	rsMRI	Low vs High suicide risk	Acc: 0.85 AUC: 0.87 Sens: 0.86 Spec: 0.78	intra-network dysconnectivity in the sensorimotor network and inter-network dysconnectivity between the default and dorsal attention network characterized high-risk patients
Chen et al., [47]	32 BD with suicidal ideation (21/11); 18 BD without suicidal ideation (9/9)	BD	Not specified	SVM (LOOCV, 1000 permutation)	12 features from ^1^H-MRS (biochemical metabolite ratios in the bilateral prefrontal white matter and hippocampus)	Suicide ideation	Acc: 0.88 AUC: 0.90 Sens: 0.94 Spec: 0.78	The most relevant features were NAA/Cr ratios in the bilateral WM, mI/Cr ratios in the bilateral WM, and NAA/Cr ratios and Cho/Cr ratios in the left hippocampus
Zhong et al., [80]	30 BD II with suicide attempts (23/7); 38 BD II without suicide attempts (21/17); 35 HC (19/16)	BD II	Unmedicated	SVM (10-folds CV)	Dynamic functional network connectivity based on rsMRI	Suicide attempts	Acc: 0.75 AUC: 0.83 Sens: 0.70 Spec: 0.79	Decreased dynamic functional connectivity variability between the left putamen and the right postcentral gyrus was found in non-suicidal compared to suicidal BD II and HC.
Xu et al., [60]	51 MDD with suicidal attempts (39/12); 74 MDD with suicidal ideation (49/25); 48 MDD without ideation/attempts (28/20); 38 HC (25/13)	MDD	Not specified	SVM (LOOCV)	Dynamic functional network connectivity based on rsMRI (>200 features)	Suicide attempts and ideation	MDD suicide attemps vs NS Acc: 0.80 AUC: 0.88 Sens: 0.88 Spec: 0. 74 MDD suicide ideation vs NS Acc: 0.75 AUC: 0.78 Sens: 0.78 Spec: 0.74MDD attempts vs ideation Acc: 0.68 AUC: 0.74 Sens: 0.68 Spec: 0.71	The features that contributed to stratifying MDD patients with diverse suicide risk levels mainly involved the visual-related and DMN-related inter-network connectivity within the weakly connected state.
Zheng et al., [108]	52 MDD with suicidal attemps (40/12); 61 MDD without suicidal attempts (36/25); 98 HC (49/49)	MDD	Not specified	XBoost	Sociodemographic, clinical and cognitive features (total: 20 features)	Suicide attempts	Acc: 0.71 AUC: 0.82 Sens: 0.6 Spec: 0.79 PPV: 0.69 NPV: 0.71	Adding cognitive information significantly increased model prediction; the most important feature was HAMD-24 score
Shin et al., [70]	83 MDD (64/19); 83 HC (69/14)	MDD	Not specified	Naive Bayes classifier (5-folds CV)	Sociodemographic and text-based	High vs low-risk suicide (based on the MINI interview)	Acc: 0.75 AUC: 0.80 Sens: 0.82 Spec: 0.65	When predicting suicide, only the ensemble analyses (namely, sociodemographic + text) resulted in significant prediction. Demographic alone: AUC 0.5 Text alone: AUC 0.64
Miranda et al., [36]	38807 PTSD patients	PTSD	Not specified	RNN	EMRs, including sociodemographic, clinical and lab features (>100 features)	Suicide-related events within 3 months	AUC:0.92	Lab tests (i.e., glucose, glucose urine, chloride, hemoglobin (HGB), hematocrit, mean corpuscular volume, white blood cell, neutrophils, potassium, INR, calcium, mean platelet volume) combined with medications and diagnoses can enhance the prediction of suicide in PTSD patients.
Zelkowitz et al., [32]	3166 (1789/1377)	Mixed diagnoses (not specified)	Not specified	RF, CART (10-folds CV)	>700 demographic and clinical features	Nonfatal suicide attempt within 30 days	RF AUC: 0.86 for men AUC: 0.86 for women CART AUC: 0.79 for men AUC: 0.81 for women	Women: Histories of self-poisoning, substance-related disorders, and eating disorders were important predictors. Men: Self-poisoning, substance-related disorders, and severe stress reactions were among the most important variables.
Tubío-Fungueiriño et al., [52]	127 (68/59)	OCD	71 AD 56 AD + AP	LDA, SVM, SVR (4-folds CV)	28 Clinical features	Suicidal thoughts	Sens: 0.79 Spec: 0.88	The most relevant features for prediction of suicidal thoughts are: trust, OCD onset, aggressive, previous YBOCS, diagnosis, OCD years, magical thinking, support, previous depression, contamination, sexual, hours info, recreation, and fear.
Yang et al., [54]	59 MDD with suicidal ideation (42/17); 22 MDD without (10/12); 60 HC (33/27)	MDD	Not specified	SVM	Six features functional connectivity between five regions with amygdala (rsMRI)	Suicidal ideation	Acc: 0.84 AUC: 0.82	The reported area that contributed to the prediction are: caudate, superior temporal gyrus, middle temporal gyrus, postcentral gyrus an their connection with amygdala
Nock et al., [37]	1818 (802/1016)	Psychiatric patients presenting in an ED	Not specified	SuperLearner stacked generalization ML method (10-folds CV)	Patient self-report (101 features), EHR, clinician prediction	Suicide attempt within 1 and 6 months	AUC for Patient self-report + EHR + clinician prediction: 0.78 for 1 month prediction; 0.78 for 6 months prediction	The prediction improved using a combination of patient self-reports and EHR data. The most important predictors resulted: past year suicide, past year suicide plan, >2 lifetime suicide attempts.
Shao et al., [58]	113 (92/21)	Late-life MDD	SSRI:42 SNRI: 21 Other AD: 41 Other drugs (AP, benzodiazepines etc): 90	SVM (LOOCV)	Clinical, sMRI and rsMRI features	Suicide attempts and suicide ideation	Acc: 0.68–0.85 Sens: 0.57–0.87 Spec: 0.57–0.91	The MRI features significantly increased classification performance of suicidal thoughts and actions over that based on clinical and suicide questionnaire variables. Most relevant features: onset age, Parietal, VLPFC/OFC, DMPFC, Precuneus, and DLPFC GMV.
Kim et al., [76]	44 (19/25) high-risk; 80 (34/46) low-risk adolescents	Adolescents with a psychiatric diagnoses (5 psychotic, 40 mood disorders, 42 ANX, 16 ADHD, 35 others)	Not specified	LR, RF, ANN, SVM, XGB (10-folds CV)	256 clinical and sociodemographic features	Low vs hig risk, based on suicide scale of the PAI-A	LR Acc: 0.89 Sens: 0.77 Spec: 0.96 RF Acc: 0.89 Sens: 0.92 Spec: 0.88 ANN Acc: 0.78 Sens: 0.77 Spec: 0.79 SVM Acc: 0.89 Sens: 0.85 Spec: 0.92 XGB Acc: 0.86 Sens: 0.92 Spec: 0.83	Most relevant features from the PAI-A contributing to the predictions: anxiety and anxiety-related scores; depression; nonsupport in social life; treatment rejection.
Ji et al., [74]	44 (34/10) 48 (28/20) MDD with suicide; MDD without suicide; 51 (28/23) HC	MDD	Not specified	SVM, AdaBoost, NB (10-folds CV)	105 clinical variables from clinical questionnaires (BDI, BSI, BHS, TDPPS, TEPS, STAI, BIS)	Suicide	SVM Acc: 0.88 Sens: 0.87 Spec: 0.89 AdaBoost Acc: 0.82 Sens: 0.87 Spec: 0.78 NB Acc: 0.82 Sens: 0.75 Spec: 0.89	Clinical questionnaires features selected with LR proved to be able to classify the groups with good accuracy. Among the most used features: pain avoidance.
Li et al., [83]	235 Recent suicide MDD (170/65); 111 Long-time suicide MDD (64/47); 1372 no suicide MDD (896/476)	First-episode MDD	Untreated	Gradient-boosted decision trees (5-folds CV)	Clinical, laboratory/biological, and demographic variables (a total of 65 variables)	Recent and long-time suicide	Recent: Acc: 0.87 Sens: 0.59 Spec: 0.61 Long-time: Acc: 0.88 Sens: 0.83 Spec: 0.50	Most relevant features for recent suicide: higher score on excitement, hostility, HAMA, HAMD and higher FT4. Most relevant features for recent suicide: single status, higher score on HAMA and Hostility, higher Low-density lipoprotein cholesterol and lower BMI.
Jiang et al., [29]	2774 (1168/1606)	MDD	Not specified	RF (10-folds CV)	Sociodemographic and clinical variables, assessed in a time-depended manner (total of 780 features for men and 803 for women)	Suicide	Men AUC: 0.70 Women AUC: 0.75	Most important predictors for men: prescriptions of hypnotics and anxiolytics, poisoning diagnoses, prescriptions for analgesics and antipsychotics, and alcohol related disorders. Most important predictors for women: receipt of state pension, prescriptions for psychiatric medications (anxiolytics and antipsychotics), poisoning, prescriptions of analgesics and hormonal contraceptives for systemic use (a marker of relationship status).
McMullen et al., [31]	571 (365/191/15) non-suicide; 20 (16/4) suicide	Psychiatric patients (298 MDD, 80 BD, 45 Anx, 43 SKZ, 64 PTSD, 34 Others)	Not specified	RF, LR, gradient-boosted trees	49 clinical features from the SCI and suicidal ideation	Suicide at 1 month follow-up	RF AUROC: 0.88 Balanced Acc: 0.72 LR AUROC: 0.83 Balanced Acc: 0.58 Gradient boosted trees AUROC: 0.90 Balanced Acc: 0.77	The model which performed the best was the combination of the SCI features and current suicidal ideation; however, the increase in the AUROC was not significant, therefore reinforcing the view that suicidal ideation should not be considered a requirement.
Coley et al., [35]	1518968 (563074/955894)	Psychiatric outpatients (74% MDD, 13% BD, 4% SKZ, 9% other)	Not specified	LR, RF (10-folds CV)	EHR from single visits	Suicide at 90-days	RF AUC: 0.85 LR AUC: 0.85	Logistic regression and random forest models using a person-level split performed well, accurately estimating prospective discrimination and classification.
Chen et al., [59]	33 (30/3) MDD with suicide attempts, 41 (23/18) MDD with suicide ideation, 54 (31/23) MDD without attempts or ideation, 58 (51/7) HC	MDD	Not Specified	CNN (5-folds CV)	Diffusion-MRI (3 T)	Suicide and suicidal ideation	AUC: 0.54–0.96 Acc: 0.53–0.91 Sens: 0.31–0.95 Spec: 0.72–0.96	Generalized q-sampling imaging- based isotropic value of the orientation distribution function increased the prediction and seemed to possess a gradient pattern moving from ideation to suicide attempt.
Liu et al., [82]	69 (39/30) MDD with suicide; 58 (28/30) MDD without suicide; 50 (26/24) HC	MDD	Not specified	SVM	Network and nodal properties form DTI MRI	Suicide	AUC: 0.79	The differential structural network connections involved the superior longitudinal fasciculus and the corpus callosum and the feeder serves as a predictor for distinguishing MDD with suicide from MDD without.
Nordin et al., [75]	75 (42/33)	MDD	Not specified	LR, decision tree, SVM, naïve Bayes, k-nearest neighbors, RF, bagging and voting (3-folds CV)	36 clinical and demographic features	Suicide	Acc: 0.79–0.92 AUC: 0.65–0.87 Sens: 0.86–0.92 Spec: 0.50–0.60 PPV: 0.79–0.89 NPV: 0.72–0.76	Bagging and voting were the most effective algorithms. Previous history of suicide, suicidal ideation, race, religion, depression severity and medical problems resulted the most important features.
Nestsiarovich et al., [64]	529359 patients with BD (301735/227624)	BD	Not specified	Tree-based XGboost34 (Balanced-data-model), LR, RF, decision tree, linear SVC (5-folds CV)	190,919 clinical covariates including age, sex, BD episode index visit characteristics, comorbid mental and physical conditions, medication prescriptions, mental health procedures ER admission in the last 1 year	Self-harm	AUC: 0.99 Sens: 0.93 Spec: 0.98	Higher risk than lithium: tri/tetracyclic antidepressant+SGA, FGA + MSA, FGA, SNRI + SGA, lithium+MSA, and lithium+SGA. Lower risk: lamotrigine, valproate, risperidone, aripiprazole, SNRI, SSRI, “No drug”, bupropion, and bupropion+SSRI. Psychotherapy alone (without medication) had a lower self-harm risk than no treatment.
Jiang et al., [29]	1205 Psychiatric patients	Mixed diagnoses (SUD, SKZ, mood disorders; anxiety, personality disorders)	Not specified	Classification trees (10-folds CV), RF (2-folds CV)	509/422 Clinical and demographic feature including diagnoses, surgeries, prescribed medications	Suicide in the 30 days after psychiatric discharge	AUC 0.70 for men AUC 0.72 for women	For men: anxiolytics and drugs interacted with other characteristics in the risk profiles (e.g., alcohol-related disorders, hypnotics and sedatives). In women, interaction between recurrent major depression and other characteristics (e.g., poisoning, low income).
Adams et al., [73]	15,953 patients with SUD (5745/10208)	SUD	Not specified	Classification tree, RF (10-folds CV)	2563 social and demographic information, mental and physical health diagnoses, surgeries, medications, and poisonings	Suicide attempts	Classification tree AUC: 0.75 RF AUC: 0.77	Among men, most important variables: reaction to severe stress, adjustment disorders, drugs used, age 30+, and prior poisoning. Among women, most important predictors: prior poisonings and reaction to stress and adjustment disorders.
Cusick et al., [50]	600 psychiatric patients (370/230)	patients at risk for suicidal ideation (80% with a diagnoses of MDD)	Not specified	SVM, CNN, Naive Bayes classifier	NLP approach to label the training and validation notes (18815 notes)	Current suicidal ideation	SVM Acc: 0.89% AUC: 0.93 CNN Acc: 94% AUC: 0.94	The CNN model outperformed all other methods on documents with “current” suicidal ideation. When applied to a random subset, the algorithm classified 23 for “current” suicidal ideation, of which 87% were truly indicative via manual review.
Bohaterewicz et al., [69]	39 SKZ patients (14/25), 20 HC (10/10)	SKZ	Not specified	GB, LASSO, LR, RF, SVM (5-fold CV)	rsMRI measures: ReHo, ALFF, fALFF, and FC.	Suicide risk measured with SBQ-R	Best performance (LASSO): AUROC 0.76 Acc: 70%	The best performance was reached for the LASSO applied to FC.
Machado et al., [89]		MDD	Not specified	Elastic-net regularization, RF, ANN (10-folds CV)	Stressful life events, and sociodemographic variables	Suicide attempts	AUC: 0.89 Acc: 81.64% Spec: 85.86% Sens: 77.42%	Previous suicide attempt, borderline personality disorder, and hospital admission for depressive symptoms were the most predictive features.
Edgcomb et al., [38]	1628 women with MDD, BD, and chronic psychosis	MDD (46%), BD (16%), psychotic disorder (51%)	AD (71%) Anxiolytic (59%) AP (45%) Stabilizers (10%)	Classification tree algorithm (10-folds CV)	420 electronic health record including: medical comorbidity, history of pregnancy-related mental illness, age, and history of suicide-related behavior	Risk factors of suicide attempt and self-harm	AUC: 0.73 Acc: 0.84 Sens: 73.4 Spec: 84.1	Predictors included medical comorbidity, history of pregnancy-related mental illness, age, and history of suicide-related behavior.
Iorfino et al., [63]	1962 young people presenting at mental health services (1184/778)	Mixed diagnoses (32% MDD; 32% Anxiety; 4% BD; 2% psychosis)	Not specified	AUCRF, Boruta, Lasso regression, Elastic-net regression, BART, LR (10-folds CV)	37 demographic and clinical variables	Self-harm at 6 months after initial presentation	AUROC: 0.74–0.76 AUPCR: 0.31–0.35 Sens: 0.67–0.75 Spec: 0.68–0.72 PPV: 0.31–0.32 NPV: 0.91–0.93	The strongest predictors were: history of self-harm, age, social and occupational functioning, sex, BD, psychosis-like experiences, treatment with antipsychotics, and a history of suicide ideation.
Zhu et al., [79]	37 BD II depressed with past suicide (27/10); 53 (33/20) BD II without past suicide (33/20); 64 HC (34/28)	BD II	AD (38%) AP (12%) Stabilizers (6%) Stimulants (8%)	SVM (5-fold CV, 1000 permutations)	significantly different frontolimbic rsFCs between the SA and NSA groups for classification analysis	Past attempts	Acc: 84%	Top predictors: frontolimbic rsFCs.
Edgcomb et al., [38]	3091 patients with MDD, BD, and psychotic disorders (1628/1463)	MDD (43%), BD (16%), psychotic disorders (55%)	AD 67% AP 47% Anxiolytic 59% Stabilizers 9%	CART (10-fold CV)	22 categories (for a total of 355 predictors) from electronic health record	Hospital readmission for suicide attempt or self-harm in 1 yr	Acc: 79.66% AUC: 0.86 Sens: 81.9% Spec: 79.7%	Incidence of suicide-related behavior highest after general non-psychiatric hospitalizations. Predictor combinations, rather than single risk factors, explained the majority of risk.
Hong et al., [53]	41 Suicide attempters (28/13) and 25 suicide ideation (18/7) young with MDD	MDD	AD 54% AP 4% Stabilizers 3%	SVM - Recursive Feature Elimination (LOOCV)	142 features including sMRI regions together with age, sex, HAMD score, and intracranial volume	Attempters vs ideation without attempt	BAC 78.59% Sens: 73.17% Spec: 84.0% PPV: 88.24% NPV 65.63%	Right lateral OFC thickness, left caudal anterior cingulate thickness, left fusiform thickness, left temporal pole volume, right rostral anterior cingulate volume, left lateral orbitofrontal thickness, left posterior cingulate thickness, right pars orbitalis thickness, right posterior cingulate thickness, and left medial orbitofrontal thickness were the 10 top-ranked classifiers for suicide attempt.
Parghi et al., [30]	591 high-risk psychiatric inpatients (381/195)	Mixed diagnoses (MDD 50% Anxiety 8% BD 13% SKZ 7% PTSD 10% OCD 0.2%)	Not specified	LR, RF, gradient boosting	49 features from the Suicide Crisis Inventory	Suicide attempt at a one‐month follow‐up	RF AUROC 87.8% Gradient Boosting AUROC 89.4%	The enhanced bootstrap approach considerably outperformed the other sampling approaches, with RF algorithms performing best in predicting positive cases of near-term suicidal behavior.
Chen et al., [33]	126,205 psychiatric inpatients and outpatient (68,151/58,054)	Mixed diagnoses (MDD 17% BD 8% SKZ 6% SUD 13% Anxiety 20% Borderline 4% ADHD 12%)	Not specified	Ensemble learning that combined predictions from elastic net penalized LR, RF, GB, and a NN (10-fold CV)	425 predictors covering demographic, SES, electronic medical records, criminality, family history of disease and crime	Suicide attempt/death within 90 and 30 days	AUC: 0.89 (30 days) AUC: 0.88 (90 days)	Top predictors: Intentional self-harm past 1 year, unplanned psychiatric visit past 1 to 3 months, diagnosis of borderline personality disorder, MDD, antidepressants, anxiolytics, benzodiazepines, and antipsychotics.
Dai et al., [68]	78 MMD (28/50)	MDD	Medication-naïve for at least two weeks	ISODATA clustering algorithm	fMRI connectivity pairs with significant difference over the well-defined groups	Suicide risk stratification	NA	The functional connectivity, locating mostly within the frontal-temporal circuit and involving the default mode network, were integrated to discriminative the gradual susceptibility of suicidal.
Fan et al., [57]	205 suicide (139/66) and 2963 non-suicide patients (2275/688)	Both PTSD and BD	Lithium 8%	LR, RF, decision tree, K-nearest neighbors, Naïve Bayes, SVM (5-fold CV)	90 features from EMR including: demographic data, number of emergency department visits and diagnoses, medication usage within one year	Suicide ideation, attempts, and deaths	Best performance (RF) TPR: 83% PPV: 91% NPV: 98%	The use of Aripiprazole, Levomilnacipran, Sertraline, Tramadol, Fentanyl, or Fluoxetine, a diagnosis of autistic disorder, schizophrenic disorder, or SUD were strong predictors.
Obeid et al., [62]	835 intentional self-harm and 1670 HC	Intentional self-harm	Not specified	Naïve Bayes, decision tree, RF, SVM, multilayer perceptron, CNN, long short-term memory with randomly initialized word embeddings.	Text from clinical notes	Concurrent and future self-harm	Best model: CNN Concurrent self-harm, AUC: 0.98 Future self-harm AUC: 0.88	The AUC for the CNN on the phenotyping task, that is, the detection of intentional self-harm in clinical notes concurrent with the events was 0.999, with an F1 score of 0.985.
Agne et al., [88]	959 OCD patients (546/413)	OCD	Not specified	Elastic net (10-CV)	89 features from demographic and clinical variables	Suicide attempts	AUC: 0.95 Sens: 84.61% Spec: 87.32% BAC: 85.97 PPV: 44.89% NPV: 97.89%	Relevant predictors: previous suicide planning, previous suicide thoughts, lifetime depressive episode, and intermittent explosive disorder.
Haines-Delmont et al., [67]	80 Inpatients (45/35)	Mixed diagnoses (Not specified)	Not specified	RF, LR, SVM, K-nearest neighbors (10-fold CV)	172 features from sleep data, journal entries, data usage, mood, and app activity statistics	Low vs high suicide risk based on C-SSRS	K-nearest neighbors Acc: 68% AUC: 0.65 RF Acc: 60% LR Acc: 59% SVM Acc: 57%	K-nearest neighbors (*k* = 2) with uniform weighting and the Euclidean distance metric emerged as the most promising algorithm.
Kessler et al., [87]	391.018 psychiatric patients from US veterans	Mixed diagnoses (psychotic disorders, mood disorders, personality disorders)	Not specified	Ensemble learning, from SVM, RF, NN, LR, elastic net, Bayesian additive regression trees	57 features from EHR, including history of suicidal behavior, psychopathological risk factors, socio-demographic, physical disorders, medications	Suicide death up to 12 months after psychiatric hospitalization	AUC: 0.79–0.82	Variable importance analysis shows that 51.1% of model performance is due to psychopathological risk factors, 26.2% to social determinants of health, 14.8% to prior history of suicidal behaviors, and 6.6% to physical disorders.
Roglio et al., [99]	689 cocaine-use disorder (442/247)	Cocaine-use disorder	Not specified	Descriptive Poisson regression and predictive RF (5 and 10-folds CV)	57 Features from sociodemographic, ASI-6, the SCID-I and the CTQ	Suicide attempts	For men: AUC: 0.68, Acc: 0.66, Sens: 0.82, Spec: 0.50, PPV: 0.47 NPV: 0.84 For women: AUC: 0.73, Acc: 0.71, Sens: 0.71, Spec: 0.71, PPV: 0.71 NPV: 0.71	This model identified several variables as important predictors, mainly related to drug use severity.
Senior et al., [66]	57 patients with severe mental illness (SKZ spectrum and BD) (23/34)	Mixed diagnoses (SKZ spectrum disorder and BD spectrum)	AD: 33% AP: 89%	Named entity recognition based on NN	EHR (free-text): history of violence, self-harm, medication, formal education, benefits recipient, drug/alcohol, parental suicide, psychiatric admission	Extracting concepts related to predictors of suicide in the OxMIS tool	Precision 0.77	The concept with the best precision and recall was medication and the weakest were suicide, and drug/alcohol use disorder.
Ge et al., [51]	1994 MDD patients with or without suicidal ideation (1377/617)	MDD	Not specified	NN (10-folds cross training)	31 predictors from demographic, clinical, and biological variables	Suicidal ideation	AUC: 0.76 Acc: 70.08% Sen: 70.68% Spec: 67.09%	The most relevant predictor variables included free thyroxine, the total scores of HAMD, vocational status, and free triiodothyronine.
Weng et al., [49]	41 MDD with suicidal ideation (23/18); 54 MDD without suicidal thoughts (31/23); and 58 HC (51/7)	MDD	Not specified	CNN-based autoencoder model, XGB, LR (5-folds CV)	25,168 features extracted from diffusion brain imaging, containing generalized FA, isotropic value of the orientation distribution function, and normalized quantitative anisotropy maps	Suicidal ideation	CNN ACC: 85% Spec: 92% Sens: 75% AUC: 0.94 LR ACC: 80% Spec: 92% Sens: 62% AUC: 0.93	The best pattern of structure across multiple brain locations can classify suicidal ideates from non-suicidal and HC with a prediction accuracy of 85%, a specificity of 100% and a sensitivity of 75%.
Tasmim et al., [105]	189 SKZ patients (76/113)	SKZ	Not specified	LR, RF, classification tree (10-folds CV)	23 items from the LEI-2 scale	Suicide attempts	LR: Acc: 62% Sens: 38% Spec: 77% PPV: 50% NPV: 67% RF: Acc: 62% Sens: 15.3% Spec: 93.2% PPV: 57.9% NPV: 64.1% Classification tree: Acc: 69% Sens: 36% Spec: 89% PPV: 67% NPV: 69%	The items “suffering from mental illness” and “sexual molestation” were classified as highly important in all three models.
Kumar et al., [61]	10,120,030 patients with major psychiatric diagnoses (6,780,420/3,339,610)	Mixed diagnoses (SKZ, schizoaffective disorder, BD, and MDD)	Not specified	Tree-based XGboost (Balanced-data-model), LR, RF, decision tree, linear SVC (5-folds CV)	185,234 unique clinical covariates (including patient age, sex, meta-visit start year, and nine feature classes: Manually curated, Procedure, Condition, Drug, Billing Code Position, Device, Observation, Measurement, and Ancestor terms.)	Self-harm	Acc: 0.94–0.96 AUC: 0.65–0.99	Self-harm undercoding was higher in male than in female and increased with age. Substance abuse, injuries, poisoning, asphyxiation, brain disorders, harmful thoughts, and psychotherapy were the main features.
Bhak et al., [106]	56 MDD with suicide attempts (30/26); 39 MDD without attempts (18/21); 87 HC (44/43)	MDD	AD 93% for attempters; 95% for non-attempters	RF	69 methylated sites from whole genome methylome, after feature selection (LOOCV)	Suicide attempts	Acc: 0.92 Sens: 0.98 Spec: 0.85 PPV: 0.90 NPV: 0.97	RF classifiers showed good accuracies in distinguishing attempters.
Xu et al., [41]	2323 patients with self-harm (1163/1160) and 46,460 inpatients controls (23,260/23,200)	Self-harm (Not specified)	Not specified	Patient embedding method, Dx2Vec (Diagnoses to Vector) followed by NN	19 features from comorbidities	Risk prediction at 12-month	Precision 0.54 Sens: 0.72 for positive cases	Dx2Vec-based model outperforms the baseline deep learning model in identifying patients who would self-harm within 12 months.
Peis et al., [48]	1023 patients (662/361)	Mixed diagnoses (Mood disorder 23%, anxiety disorders 53%)	Not specified	Recurrent NN	117 features from EMA in combination with traditional EHR	Suicide ideation	Acc: 95% AUC 0.94	Addition of EMA records boosts the prediction of suicidal ideation diagnosis from 48.13% obtained exclusively from EHR-based state-of-the-art methods to 67.78%.
Gosnell et al., [81]	423 psychiatric inpatients (Not specified)	Mixed diagnoses (mood, anxiety, personality, and SUD)	Not specified	RF (LOOCV)	Structural (316) and resting-state functional connectivity (8256) measures	Suicide attempts	Sens: 79.4% Spec: 72.3%	Altered resting-state functional connectivity features from frontal and middle temporal regions, as well as the amygdala, parahippocampus, putamen, and vermis were found to generalize best.
Carson et al., [109]	73 adolescents psychiatric inpatients (45/28)	Mixed diagnoses (Not specified)	Not specified	RF (5-folds CV)	Notes from electronic medical records	Suicide attempts in the past year	AUC: 0.68 Acc: 0.47 Sens: 0.83 Spec: 0.22 PPV: 0.42 NPV: 0.67	The terms mostly highly associated clustered around terms related to suicide, family members, psychiatric disorders, and psychotropic medications.
Fernandes et al., [84]	Not specified	Mixed diagnoses (Not specified)	Not specified	SVM after NLP	500 notes from correspondence and questionnaires	Suicide attempts Suicide ideation	Attempts PPV: 91.7% Sens: 87.8% Ideation PPV: 82.8% Sens: 98.2%	Good performance of the two classifiers in the evaluation study suggest they can be used to accurately detect mentions of suicide ideation and attempt within free-text documents.
Jordan et al., [40]	218 psychiatric patients with previous attempts (109/109)	Mixed diagnoses (Psychosis 10%; MDD 79%; BD 11%; personality disorder 73%; alcohol/SUD 62%)	Not specified	IROC analysis, LR	Variables from sociodemographic and clinical scales	1-year suicide attempt after discharge	AUC: 0.63 Sens: 55.77% Spec: 69.11% PPV: 43.28% NPV: 78.70%	The cross-validated IROC, but not logistic regression, predicted attempts. Furthermore, participants who made definite plans and underwent extensive preparation were at highest risk.
Oh et al., [110]	573 anxiety or MDD patients (306/267)	Anxiety and MDD	Not specified	NN	41 variables: 31 psychiatric scales and 10 sociodemographic variables	Lifetime, past year, past month suicide attempts	1-month: Acc: 93.7% Spec: 99.6% Sens: 12.8% 1-year: Acc: 90.8% Spec: 98.4% Sens: 33.8% Lifetime: Acc: 86.4% Spec: 91.2% Sens: 77.9%	Among all variables, the Emotion Regulation Questionnaire had the highest contribution, and the positive and negative characteristics of the scales similarly contributed to classification performance.
Hettige et al., [111]	345 patients with SKZ (104/241)	SKZ	Not specified	LASSO, RF, SVM, elastic net (10-folds CV)	27 sociocultural and clinical variables	Suicide attempts	Lasso AUC: 0.71 Acc: 0.67 Sens: 0.64 Spec: 0.68 PPV: 0.67 NPV: 0.66 RF AUC: 0.67 Acc: 0.66 Sens: 0.45 Spec: 0.80 PPV: 0.68 NPV: 0.60 SVM AUC: 0.70 Acc: 0.66 Sens: 0.63 Spec: 0.68 PPV: 0.67 NPV: 0.66 Elastic Net: AUC: 0.71 Acc: 0.65 Sens: 0.65 Spec: 0.65 PPV: 0.65 NPV: 0.66	The four models did not differ in pair-comparison as tested using the McNemar’s test. The weighs of the predictors differ a lot between the four models.
Setoyama et al., [46]	94 affective patients (Not specified)	MDD	Both medicated and non-medicated patients	LR, SVM, RF	123 metabolites from metabolome analysis	Suicidal ideation	AUC 0.60–0.79	Kynurenine, Xanthurenate, Xanthosine, Citrate and Alanine correlated with suicide.
Pestian et al., [112]	130 suicidal patients, 126 non-suicidal patients with mental illness, and 123 controls (Not specified)	Mixed diagnoses (Not specified)	Not specified	SVM (LOOCV)	Linguistic and Acoustic Features extracted from open-ended questions	Suicide attempts	Suicidal vs HC ROC: 0.92 Suicidal vs non-suicidal Patients: ROC: 0.82 Suicidal vs All: ROC: 0.87	By combining linguistic and acoustic characteristics, subjects could be classified into one of the three groups.
Barros et al., [65]	707 mental health patients (564/143)	Mixed diagnoses (MDD 53% BD 18% Anxiety 10% Adjustment 10% Dysthymia 1% Others 8%)	Not specified	CART, k-nearest neighbor, RF, AdaBoost, NN multilayer perceptron, SVM (10-folds CV)	343 sociodemographic and clinical variables	Suicide risk (current suicidal behavior – attempts o ideation)	SVM Acc: 0.78 Sens: 0.77 Spec: 0.79 CART Acc: 0.72 Sens: 0.71 Spec: 0.74 RF Acc: 0.78 Sens: 0.78 Spec: 0.77 AdaBoost Acc: 0.76 Sens: 0.75 Spec: 0.76 KNN Acc: 0.73 Sens: 0.74 Spec: 0.73	The model shows that the variables of a suicide risk zone are related to individual unrest, personal satisfaction, and reasons for living, particularly related to beliefs in one’s own capacities and coping abilities.
Cook et al., [45]	1453 self-harming patients (944/509)	Self-harm (Not specified)	Not specified	NLP-Based Machine Learning (linear classifier)	Open-ended question from a medical app	Suicidal ideation	Sens: 0.56 Spec: 0.57 PPV: 0.61	The top ten words associated with suicidal ideation were conté (I told), monotona (monotony), Equasim (Ritalin), acosado (harassed), trabajamos (we work), raza (race), aseos (restrooms), resfriado (congested/sick), pronuncio (I pronounce), and rechaza (rejects).
Kessler et al., [44]	Veterans with a mental disorder diagnosis (Not specified)	Mixed diagnoses (Not specified)	Not specified	naive Bayes, RF, SVR, elastic net penalized regression	Nearly 1000 variables from sociodemographic, career, medical records	Suicide deaths after a psychiatric visit	AUC: 0.61–0.75	An elastic net classifier with 10–14 predictors optimized sensitivity (45.6% of suicide deaths occurring after the 15% of visits with highest predicted risk).
Walsh et al., [4]	5167 patients with a self-injury diagnosis (2432/2706/29)	Self-harm (Not specified)	Not specified	RF	>200 features Demographic and clinical	Suicide attempts	AUC: 0.84	Recurrent depression with psychosis, SKZ, and schizoaffective disorder were ranked highly in importance.
Morales et al., [56]	707 affective patients (564/143)	Mixed diagnoses (MDD 53% BD 18% Anxiety 10% Adjustment 10% Dysthymia 1% Others 8%)	Not specified	Decision tree	345 variables from clinical scales	Suicidal behavior (attempt or ideation)	Acc: 0.67–0.71 Spec: 0.65–0.79	Suicide risk configuration variables: thought about taking one’s life, frequent headaches, dissatisfied or not very satisfied with life, empty inside, and not feeling happy, depressive lifestyle.
Passos et al., [91]	144 patients with mood disorders (Not specified)	MDD or BD types I or II	Not specified	LASSO, SVM, RVM (LOOCV)	17 clinical and demographic variables	Suicide attempts	RVM AUC: 0.77 BAC: 72% Sens: 72% Spec: 71% PPV: 51% NPV 86% SVM AUC: 0.65 BAC: 65% Sens: 58% Spec: 71% PPV: 46% NPV 80% LASSO AUC: 0.73 BAC: 68% Sens: 56% Spec: 80% PPV: 55% NPV 81%	All algorithms had significant prediction accuracy (64.7%–72%). RVM yielded the highest result. The most relevant predictor variables included: number of previous hospitalizations for depression, psychosis, cocaine dependence, PTSD.
Levey et al., [43]	51 women with psychiatric disorders	Mood disorders and SKZ	Not specified	Convergent Functional Genomics followed by ROC curve	50 genetic derived biomarkers combined with clinical scales	Suicidal ideation and future hospitalization for suicide	AUC: 0.94 for the genetic predictors	50 validated biomarkers predicted future hospitalizations due to suicidality. Best predictors: BCL2, GSK3B, and PIK3C3, circadian clock genes (PER1 and CSNK1A1), docosahexaenoic acid signaling pathways genes.
Niculescu et al., [42]	37 males with psychiatric disorders	Mood disorders (BD 15, MDD 7), SKZ (6), schizoaffective (4), PTSD (3), Others (2)	mood stabilizers; AD; AP; benzodiazepines; and others (percentages not available)	Convergent Functional Genomics followed by ROC curve	76 genetic derived biomarkers	Suicidal ideation and future hospitalization for suicide	Transdiagnostic AUC: 0.92; BD AUC: 0.98	The best individual biomarker across psychiatric diagnoses for predicting suicidal ideation was SLC4A4.
Kessler et al., [39]	Soldiers with a psychiatric diagnoses and hospitalization (Not specified)	Mixed diagnoses (Not specified)	Not specified	Elastic net (10-folds CV)	421 predictors from demographic, career and clinical variables	Suicides in the 12 months after hospital discharge	AUC:0.71–0.84	The strongest predictors included sociodemographics (male sex and age), criminal offenses, prior suicidality, prior psychiatric treatment, and diagnosis.
Tran et al., [34]	7578 mental health patients (3842/3736)	Mixed diagnoses (Not specified)	Not specified	Non-negative restricted Boltzmann machines followed by SVM (10-folds CV)	EMR	Suicide risk stratification 3 months	NA	The derived representation not only shows clinically meaningful feature grouping but also facilitates short-term risk stratification.
Poulin et al., [113]	70 HC; 69 suicide attempters; 70 psychiatric patients (Not specified)	Mixed diagnoses (Not specified)	Not specified	Bags of words by Meta-Optimizing Semantic Evolutionary Search (5-folds CV)	Words from clinical records (27–77 clinical records for each subject)	Suicide attempts	Acc: 65%	For single-word models, the accuracy was 59%, and scores for individual candidate models ranged from 46–65%. For pre-selected word pairs, the individual model scores ranged from 52–69%, with an average of 64%. The combined Cohorts ‘1v2v3 classifier’ had a peak performance of 67%, and an average performance of 65%.
Delgado-Gomez et al., [114]	345 suicide attempters, 150 psychiatric inpatients and 384 HC (Not specified)	Mixed diagnoses (Not specified)	Not specified	Linear discriminant analysis, Fisher linear discriminant analysis, boosting, SVM	30 features from the BIS-11 and 77 from the IPDE-SQ	Suicide attempts	SVM Acc: 79% Sens: 66% Spec: 87% Boosting Acc: 77% Sens: 64% Spec: 85% Linear discriminant Acc: 77% Sens: 65% Spec: 85% Fisher linear Acc: 78% Sens: 65% Spec: 88%	The most discriminative BIS-11 and IPDE-SQ items are “I am self controlled” (Item 6) and “I often feel empty inside” (item 40), respectively.
Lopez-Castroman et al., [71]	1349 suicide attempters (1140/209)	Mixed diagnoses (MDD 72% BD 15% Anxiety 52% Psychotic disorders 3% Eating disorder 12% OCD 5%)	Not specified	SVM (10-folds CV)	Ten clinical and demographic variables selected through Markov Blanket	Number of suicide attempts	Sens: 51–67% Spec: 68–98%	Risk of frequent suicide attempt was highest among middle-aged subjects and diminished with advancing age. Anxiety disorders significantly increased the risk. Frequent suicide attempts were linked to alcohol and SUD and more intensive treatment.
Baca-Garcia et al., [98]	277 male psychiatric patients	Mixed diagnoses (Attempters: SUD 47%, SKZ 13%, MDD 55%, BD 7%, Anxiety 32%, OCD 2%, Adjustment 5%, somatoform 2%, eating disorders 1%. Non-attempters: SUD 27%, SKZ 42%, MDD 22%, BD 10%, Anxiety 34%, OCD 24%, Adjustment 1%, eating disorders 1%.	Not specified	SVM	840 SNPs	Suicide attempts	Sens: 0.50 Spec: 0.82 positive likelihood ratio: 2.80 negative likelihood ratio: 1.64	Three SNPs of three genes (rs10944288, HTR1E; hCV8953491, GABRP; and rs707216, ACTN2) correctly classified 67% of male suicide attempters and non-attempters.
Ilgen et al., [92]	887,859 veterans with depression (Not specified)	MDD	Not specified	Decision tree based on Bayesian Dirichlet Equivalent	Clinical and demographic features	Suicide attempts	NA	Most relevant factors: a co-occurring SUD diagnosis, male sex, African American race, and psychiatric hospitalization in the past year.
Mann et al., [86]	408 patients with mood, SKZ spectrum, or personality disorders (Not specified)	Mixed diagnoses (Attempters: mood disorders, 54%; SKZ spectrum disorders, 34%; others 22%. Non-attempters: mood disorders, 58%; SKZ spectrum disorders. 37%; others 16%	Not specified	CART (LOOCV)	25 features from sociodemographic, diagnostic and clinical scales	Recent or remote suicide attempts	Recent attempt: Sens: 73% Spec: 80% PPV: 58% Remote suicide attempt: Sens: 89% Spec: 36% PPV: 44%	In equally weighted trees, a recent past suicide attempt was best predicted by current suicidal ideation and no adequate model was found for remote attempts. In unequally weighted models, recent attempters were identified by suicidal ideation and comorbid borderline personality disorder. Remote attempters were identified by lifetime aggression and current subjective depression.
Baca-Garcia et al., [72]	539 suicide attempters (343/196)	Mixed diagnoses (Mood disorders 41%, SUD 24%, SKZ 8%, eating disorders 5%, Anxiety 5%)	Not specified	RF, Forward Selection	101 clinical variables	Family history of suicide	Acc: 92.1–98.7% Sens: 78.4%–88.2% Spec: 92.5–96.6%	A classificatory model for family history of attempted suicide included the use of alcohol in the intent and family history of completed suicide.
Tiet et al., [28]	34,251 SUD veterans (33,242/1009)	SUD	Not specified	Decision tree	Demographic factors, diagnoses, and the core ASI items	30-day risk of an actual suicide attempt	Sens: 0.33–0.89 Spec: 0.42–0.87	The factors included encompass history of prior attempts, drinking to intoxication, cocaine use, first occasion of suicidal ideation, and difficulty controlling violent behavior.
Modai et al., [21]	987 psychiatric inpatients (310/677)	Mixed diagnoses (SKZ 56%, Schizoaffective 12%, MDD 3%, BD 3%, personality disorders 7%, anxiety 2%, others 18%)	Not specified	FALCON NN	20 suicide risk factors variables	Medically serious suicide attempts	Sens: 91%–94% Spec: 82%–85%	Trained FALCON, a nonlinear neural network, achieves respectable accuracy in detecting patients based on 20 suicide risk factors.
Modai et al., [21]	612 psychiatric patients (185/427)	Mixed diagnoses (SKZ 72%, schizoaffective 11%, MDD 1%, BD 3%, anxiety 0.5%, personality disorders 4%, organic syndromes 4%, others 15%)	Not specified	FALCON NN	21 features from CSRS-III	Medically serious suicide attempts	Acc: 71.5% Sens: 83% Spec: 70%	The influence of the various risk factors differed for diagnoses.
Modai et al., [115]	197 inpatients (Not specified)	Mixed diagnoses (Not specified)	Not specified	FALCON NN; Backpropagation enhanced with fuzzy logic	59 suicide-associated variables	Medically serious suicide attempts	Sens: 94 + 6.9% Spec: 69 + 6.9%	BP-fuzzy logic trained with programmer input sets, with mean + SD results of 97 + 3.4% sensitivity and 69.25 + 6.9% specificity. The second was the FALCON trained with 15 suicide-associated clinical variables where mean + SD results were 94 + 6.9% sensitivity and 69 + 6.9% specificity.
Modai et al., [93]	198 patients (Not specified)	Mixed diagnoses (Not specified)	Not specified	Backpropagation NN; LR	44 demographic and clinical variables	Medically serious suicide attempts	TSR: 91.8% PPV: 92% NPV: 95.6%	Living alone, treatment compliance, drug abuse or dependence, GAF score, non-paranoid delusions and suicide of first degree relative were highly associated.
Modai et al., [116]	161 hospitalized psychiatric patients (Not specified)	Mixed diagnoses (Not specified)	Not specified	Backpropagation NN	54–150 records	Medically serious suicide attempts	Sens: 63–71% Spec: 60–95% PPV: 30–75% NPV: 90–91%	At present, neural networks are not reliable instruments for evaluating suicidal risk due to the significant number of false positive results.

*Acc*: accuracy, AD antidepressants, *ADHD* attention deficit hyperactivity disorder, *ALFF* amplitude of low frequency fluctuations, *ANN* artificial neural networks, *AP* antipsychotics, *ASI* addiction severity index, *AUC* area under the curve, *AUCRF* area under the curve RFs, *AUROC* area under the receiving operator curve, *AUDIT* alcohol use disorders identification test, *AUPRC* area under the precision-recall curve, *AUROC* area under receiver operating curve, *BAC* balanced accuracy, *BART* Bayesian additive regression trees, *BD* bipolar disorder, *BDI* Beck depression scale, *BHS* Beck hopelessness scale, *BIS-11* Barratt’s impulsiveness scale version 11, *BSI*: Beck scale for suicidal ideation, *CART* classification and regression tree algorithm, *CNN* convolutional neural network, *CT* childhood trauma questionnaire, *CV* cross-validation, *C-SSRS* Columbia-suicide severity rating scale, *CSRS-III* computerized suicide risk scales, *DLPFC* dorsolateral prefrontal cortex, *EMA* ecological momentary assessment, *EHR* electronic health records, *EMR* electronic medical records, *FA* fractional anisotropy, *FALCON* fuzzy adaptive learning control network, *FC* functional connectivity, *FGA* first generation antipsychotic, *GAF* global assessment of functioning, *GB* gradient boosting, *HAMA* Hamilton anxiety scale, *HAMD* Hamilton depression scale, *HC* healthy control, ^1^*H-MRS* proton magnetic resonance spectroscopy, *IPDE-SQ* international personality disorder evaluation screening questionnaire, *ISODATA* iterative self-organizing data analysis techniques, *IROC* iterative receiver operator characteristic analysis, *KNN* k-nearest neighbor *LEI-2* life event inventory, *LDA* linear discriminant analysis, *LOOCV* leave-one-out cross-validation, *LR* logistic regression, *MDD* major depressive disorder, *MIN*: mini-international neuropsychiatric interview, *MSA* mood-stabilizing anticonvulsant, *NLP* natural language processing, *NN* neural network, *NPV* negative predictive value, *NSSI* non-suicidal self-injury, *OCD* obsessive-compulsive disorder, *OFC* orbitofrontal cortex, *OxMIS* Oxford mental illness and suicide tool, *PAI-A* personality assessment inventory for adolescent, *PPV* positive predictive value, *PTSD* post-traumatic stress disorder, *ReHo* regional homogeneity, *RF* random forest, *RNN* recurrent neural network *RVM* relevant vector machine, SBQ-R suicide behavior questionnaire—revised. *SCI* suicide crisis inventory, *SCID-I* structured clinical interview for DSM-IV axis I disorders, *Sens* sensitivity, *SES* socioeconomic status, *SGA*: second-generation antipsychotic, *SNP* single nucleotide polymorphism, *SNRI* serotonin and noradrenaline reuptake inhibitors, *SKZ* schizophrenia, *SNP* single nucleotide polymorphism, *Spec* specificity, *SSRI* selective serotonin reuptake inhibitors, *STAI* state-trait anxiety inventory, *SUD* substance-use disorder, *SVM* support vector machine, *SVR* support vector regression, *TDPPS* three-dimensional psychological pain scale, *TEPS* temporal experience of pleasure scale, *TPR* true positive rate, *TSR* total success rate, *VLPFC* ventrolateral prefrontal cortex, *XGB* extreme gradient boosting.

### Description of outcome employed

Regarding the predicted outcome, 41 (51%) studies used ML to predict lifetime suicide attempts (e.g., retrospective assessed past attempts), while only 16 (19.7%) longitudinally assessed the risk of suicide using future risk/attempts as an outcome. Specifically, five studies [28–32] predicted the attempts/death at 1 month after the actual evaluation, the study by Chen and colleagues [33] predicted suicide attempts at both one and 3 months from the assessment, while three studies [34–36] predicted suicide risk at three months, and Nock and colleagues [37] predicted suicide between 1 and 6 months. Three studies [38–40] predicted suicide attempts at 12 months, and one study [41] stratified suicide risk at 12 months after the actual assessment. Finally, three studies [42–44] predicted future hospitalization for suicide or future suicide attempts without defining a precise temporal window.

Moreover, 14 studies predicted suicide ideation alone [45–55] or in combination with suicide attempts [56–60]. Finally, other studies predicted self-harm [61–64], suicide risk [38, 55, 65–70], the number of suicide attempts [71], and the presence of a familiar history of suicide [72].

### Description of ML algorithms used

Regarding the number and type of ML approaches employed in the studies, 46 (57%) of the retrieved papers used a single ML algorithm, while 35 (43%) employed more than one. Among those employing more than one ML method, the average number of ML algorithms used was 3.8, with a range from 2 to 7. The most used algorithms were random forest (RF) and support vector machine (SVM), which were employed 29 times each, followed by neural networks-based approaches and decision tree-based approaches, employed 22 and 18 times, respectively. Other ML approaches were used more scarcely: elastic net eight times, Bayesian-based approaches six times, and clustering methods only four times.

Among studies adopting only one ML algorithm, neural networks were used 12 times, SVM 11, RF 5, tree-based approaches 4 times, and elastic nets three times.

In the studies that compared more than one algorithm, ML methods always performed better than LR. Moreover, RF [32, 57, 73] and SVM [74, 75] resulted among the best-performing algorithms, often with comparable results [65, 76], when compared to other methods. Finally, when present, CNN outperformed other ML methods [49, 50, 62, 77], including SVM and RF (please see Supplementary Table 4 for further details).

### Description of the sample sizes and most assessed diagnoses

Sample sizes varied substantially across studies, ranging from 37 [42] to 10,120,030 [61] individuals, with an average of 230,074.5 and a standard deviation of 1,392,637. More in detail, twelve studies (14.8%) enrolled less than 100 participants, 27 studies (33.3%) enrolled between 100 and 500 individuals, 12 studies (14.8%) between 500 and 1000, 15 studies (18.5%) between 1000 and 10,000 and the remaining ten studies (12.3%) more than 10,000 subjects. For six studies, it was not possible to retrieve the exact number of participants included in the analysis.

Given the relatively low prevalence of the event of interest (i.e., suicide), most of the samples were unbalanced in terms of the number of subjects in each group. For instance, in the studies conducted by Fan and colleagues [57] and Wang and colleagues [77], the difference in size between the suicidal group and the non-suicidal control group was tenfold (i.e., 205 subjects in the “suicide” group and 2963 in the “no suicide” group). Similarly, the difference in Xu et al. [41] was 20-fold, with 2323 patients reporting self-harm and 46,460 patients with no self-harm characteristics. It is important to note that, on the one hand, very large differences in sample size require significant corrections in the predictive algorithm (e.g., the weighting of the hyperplane for uneven group sizes), whereas, on the other hand, they reflect real data, as the prevalence of suicidal events in the assessed population is typically low.

Regarding the psychiatric diagnoses, 45 studies (55.5%) included more than one diagnosis in their sample and assessed the risk of suicide in a transdiagnostic manner, whereas 36 studies (45.5%) focused on patients with a single specific diagnosis. Not all the studies reported full details regarding the diagnostic status of the included sample, with some of them only referring to “psychiatric patients” to describe the sample.

Among reports detailing patients’ diagnosis, mood disorders were prevalent in 64 studies (79%). Specifically, major depressive disorder (MDD) was studied in 37 investigations, and bipolar disorder (BD) in 21 publications. Six studies simply reported “mood disorders” to characterize the sample. Patients affected by schizophrenia were included in 14 studies, whereas four enrolled patients diagnosed with schizoaffective disorder and five simply reported “psychosis” as a sample description. Thirteen studies focused on anxiety disorders, eight on substance-use disorders and four on obsessive-compulsive disorders.

Finally, among studies focusing on a single diagnosis, MDD was the most represented one (16 times), followed by BD, schizophrenia, and substance-use disorders represented three times each.

### Description of the number and types of features

The number of features employed in the prediction of suicidal behaviors varied considerably across studies, ranging from 10 [71] to 190,919 [64]. Specifically, 20 studies (24.7%) predicted suicide with less than 50 features, seven studies (8.6%) employed between 50 and 100 features, 11 (13.6%) between 100 and 200, ten (12.3%) between 200 and 500, and, lastly, 11 studies (13.6%) employed more than 500 features. In addition, 22 studies (27.1%) did not report the exact number of features being fed to the algorithm for suicide prediction.

As far as the feature types are concerned, the majority of the studies (54, 66.6%) used clinical and sociodemographic variables. Among these, ten studies were based on electronic health records (EHR), which are becoming an important source of data in the last few years [78].

Ten studies employed brain imaging data to predict suicide: seven studies used resting-state MRI (rsMRI) [54, 55, 60, 68, 69, 79, 80], two used both rsMRI and structural MRI [58, 81], three used diffusion tensor imaging (DTI) [49, 59, 82], and one structural MRI in combination with clinical and demographic data [53], and one single study employed measures from spectroscopy [47]. Eight studies (13.6%) analyzed the text obtained from interviews and EHR using natural language processing (NLP).

Only four studies (4.9%) focused on genetics and epigenetics features in order to predict suicide, and a single study [46] explored the predictive value of the human metabolome, employing 123 plasma metabolites, to predict suicide. Lastly, three studies [36, 51, 83] used blood biochemistry in association with clinical and sociodemographic data.

### Description of AUC and accuracy ranges

A total of 62 studies (76.5%) reported at least the accuracy or the area under the curve (AUC) of their prediction, while the remaining studies reported different metrics (e.g., positive predictive value, sensitivity F1 score [84]), also because of the methods employed (e.g., clustering and neural networks [41, 68]).

**Fig. 2:**
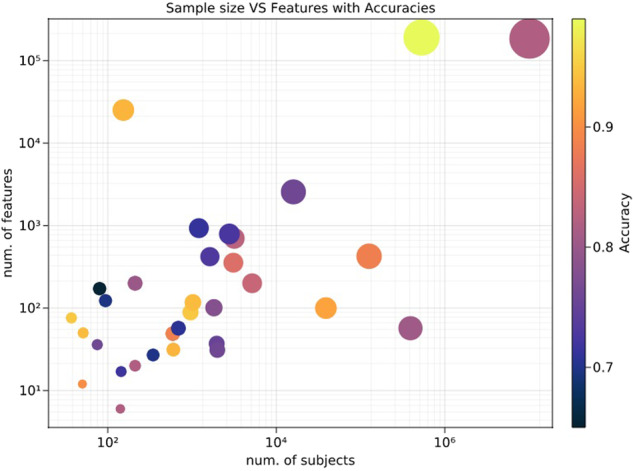
Graphical representation of the AUCs as a function of the number of features and the sample size. When the authors performed more than one analyses using the same features and sample, the highest prediction value was used for the present graph. Features number and sample size are reported in a logarithmic scale. The color bar indicates the prediction rate. Good predictions are reached even with a limited number of subjects and features. However, this graph does not hold any meta-analytic value, given the differences between the studies.

Interestingly, 87% of studies (i.e., 54 out of 62) focusing on either prediction accuracy or AUC reported values above 70% or 0.70, respectively. Specifically, eleven studies reported an accuracy between 70 and 80%, 14 between 80 and 90%, and six studies above 90%. Regarding AUC, 14 studies showed AUC between 0.70 and 0.80, 16 between 0.80 and 0.90, and eleven studies reported AUC above 0.90. The AUC of selected studies is reported in Fig. 2 as a function of sample sizes and number of features. Nonetheless, besides a few notable exceptions [38, 42, 43], no studies tested their prediction on independent validation samples. However, it is noticed that in highly unbalanced samples, the lack of an independent validation sample greatly reduces the overall generalizability. Therefore, these findings are likely to suffer from overfitting and should be regarded with caution [85].

### Most relevant features

Studies employing clinical and sociodemographic variables confirmed previous suicide risk factors. Previous suicide attempts, suicidal behaviors, or self-harm acts were among the strongest and most replicated predictors [28, 32, 33, 37–39, 61, 63, 71, 73, 75, 86–90]. Similarly, the type and severity of the psychiatric diagnosis seem to be associated with an increased risk of suicide. In detail, diagnosis and severity of MDD [4, 33, 56, 86, 88, 89, 91], psychotic features alone or accompanied by mood disorder [4, 63, 91], borderline personality disorder [33, 86, 89] and previous psychiatric hospitalizations [91, 92], ranked among the most relevant features. Moreover, also comorbidity with alcohol or substance use or abuse emerged as relevant features, irrespectively of the initial diagnosis [28, 57, 71–73, 90–93]. Interestingly, a significant effect on suicide prediction was reported for the use and dosage of psychiatric pharmacotherapy, specifically antipsychotics [33, 63, 64] and antidepressants, especially tricyclics [33, 64, 73]. Moreover, variable importance analysis in a sample of 390,000 US veterans showed that 51.1% of model performance was driven by psychopathological risk factors, 26.2% by social determinants of health, 14.8% by prior history of suicidal behaviors, and 6.6% by physical disorders [87].

In line with this result, other ML studies highlighted the importance of socio-occupational status and well-being [56, 63, 65, 87, 93]. Similarly, non-psychiatric health issues have been reported among the features able to predict suicide [38, 56, 94]; moreover, one study reported the use of commonly prescribed opioids (e.g., Fentanyl) as a relevant feature in the prediction [57].

Regarding demographic variables, sex, and age differences also emerged. Sex resulted in a significant predictor in five studies, showing either increased risk for males [39, 63, 92] or more complex relationships between biological sex and risk factors [29, 73]. Moreover, age ranked among the most predictive features in five studies [38, 39, 63, 71, 73, 94], with Lopez-Castroman and colleagues [71] also suggesting that the risk increases until middle-aged, but then tends to decrease in the elderly. Lastly, only two studies [72, 93] reported family history of suicide among the most relevant features assessed, whereas criminal or violent behavior were listed as predictive in two other investigations [28, 39].

Regarding the studies that assessed the predictive power of brain imaging data, the thickness and volume of the orbitofrontal, the anterior and posterior cingulate, and the temporal areas were selected by the algorithm as best predictors of suicide attempts in a group of young individuals and MDD patients [53], while in late-life depression sample, frontal areas and precuneus emerges as the strongest predictors [58]. Moreover, measures of functional connectivity [69] of frontolimbic [79, 81] and fronto-temporal circuits, as well as of the default mode network (DMN) [54, 68, 81], the amygdala, the parahippocampus and the putamen [54, 81], attained classification accuracies above 70%.

Regarding clinical predictors in MDD populations, Ilgen and colleagues [92] reported that co-occurring substance use, male sex, and previous psychiatric hospitalizations increased the risk of suicide. Similarly, in a more recent publication [89], hospitalization, previous suicide attempts, and co-diagnosis with a personality disorder resulted in the most relevant features to predict suicide, yielding an accuracy above 80%. Moreover, thyroxine plasma level and the severity of depression (measured via the Hamilton scale for depression - HAMD) were able to predict suicide with an accuracy of 70% [51].

In studies that involved a broader spectrum of diagnoses of mood disorders (including MDD, BD and also anxiety disorders), previous history of suicide or suicidal thoughts [56, 63], presence of psychotic features [63, 91], and socio-occupational functioning [56, 63, 65] ranked among the most important features in the prediction (all scoring above 70% accuracy). Lastly, Passos and colleagues [91] showed a significant contribution of substance use or dependence and of the number of previous hospitalizations to suicide risk, whereas Iorfino and colleagues [63] found that treatment with antipsychotics, sex, and age were relevant features in the prediction. A brief summary of the most important features is reported in Supplementary Table 5.

## Discussion

The objective of our review was to summarize the results of ML studies in predicting suicidal behaviors in psychiatric clinical populations. Although the earliest publication in our review dates back to 1998, more than half of the reports were published between 2019 and 2022, ultimately suggesting that ML approaches in psychiatry, and especially in suicide prediction, are becoming more and more frequent nowadays. It is, therefore, important to constantly update the literature evaluation in order to keep pace with an exponentially increasing field. This translates into the opportunity to critically guide the nascent field and address key gaps in the existing literature. Compared to previous literature [95], our review focused only on psychiatric samples, in order to reduce the bias given by the diagnoses in general population. When focusing on broader samples, studies tend to find the presence of a psychiatric diagnosis as one of the most predictive features. Since it is well-known that the psychiatric population are at higher risk for suicidal behaviors, using general population often does not add knowledge in suicide prevention, while on the other side might mask more subtle risk factors. Moreover, compared to previous reviews in the field [95], we gave a more in-depth analysis of predictive features and also employed two different scoring ranking especially designed for ML studies (see Supplementary materials), in order to give the most precise overview of the literature. Critically, all these aspects might serve as a starting point for future studies.

Regarding our results, most studies classified lifetime suicide attempts, and fewer assessed suicidal attempts in a follow-up time window [28–32, 38, 39, 96]. Moreover, some studies classified their sample for death by suicide [44], suicidal ideation [45, 46, 48–51, 56, 57], or risk stratification [38, 41, 65–69]. Differences in the outcomes and in the definition of risk pose a problem for the interpretation of the results, as risk factors for suicide are reported to be different from those for self-harm and suicidal ideation [1, 97]. In addition, studies also varied in terms of sample selection. Indeed, while most of the publications assessed suicide as a transdiagnostic outcome [38, 40, 63, 66, 67, 81, 98], only a few authors focused on patients with a specific diagnosis, mostly mood disorders [46, 51, 53, 58, 68, 75, 89, 92]. These differences limit the translation of the findings into clinical practice. Prediction models will likely improve prediction accuracy and inform clinical decisions if tailored not just for specific diagnostic groups but also on a dimensional approach to psychiatric disorders [16], as every diagnosis has a different and specific type of assessment and disease trajectory. This means that different patients’ groups might have different predictive features, with probable overlaps between diagnoses. Therefore, a focus on specific diagnostic groups should not divert attention from a comprehensive evaluation of the patient, given that both physical and psychiatric (especially substance abuse disorder) comorbidities proved among the most important predictive features.

Furthermore, another main issue regarding the reviewed studies is the imbalance between the prediction groups, given the low prevalence of the event of interest, with some studies including a larger control group, even tenfold bigger, than the suicidal group [41, 57, 77]. Although an imbalance is intrinsic to this kind of studies, given the prevalence of suicide in psychiatric disorders, some methods can be deployed to reduce the risk of false positive. Fan and colleagues [57] opted for an oversampling in the training phase, a procedure that creates new samples by connecting inliers and outliers from the original dataset. This technique allows the creation of dummy subjects to balance the sample, to foster the reliability of the ML analysis. Other analytical procedures to overcome the issue of imbalanced samples imply weighting of the hyperplane for uneven group sizes, selecting a specific “weight” based on the difference between the groups.

Notably, in most of the cases, the variables employed as predictors were clinical and sociodemographic [48, 57, 87]. Several of the strongest predictors in ML studies are well-known risk factors for suicide, such as previous suicide attempts, previous hospitalizations, and severity of depression [28, 38, 51, 89, 91, 94, 96, 99]. Moreover, the presence of psychosis and a higher amount of pharmacological treatments, especially antipsychotics, resulted to be highly predictive features in many investigations [4, 63, 64, 91, 100, 101]. Interestingly, also presence of psychiatric comorbidities was one of the most valuable predictive features, in particular substance or alcohol use disorders [57, 61, 71, 72, 92]. These results emphasize the importance of a comprehensive evaluation of psychiatric patients and of the burden that comorbidities represent, also given their frequent occurrence [102]. This is particularly important for the comorbid use of alcohol and drug abuse, since they can reduce compliance to treatments [103] and increase impulsive behaviors [104], which in turn may act as risk factors for suicide. Besides the well-known suicide risk factors (i.e., history of suicide attempts, hospitalizations, etc.), more subtle risk factors emerged from the reviewed studies. More in detail, comorbidities resulted in important features in different studies, suggesting that not only psychiatric comorbidities but also physical health is important. Similarly, the use of specific drugs (i.e., antipsychotics), illness severity, and psychosis seemed to be highly predictive of suicide attempts. Finally, some studies suggested that also laboratory tests, such as thyroid hormones, might play a role in predicting suicidal behaviors, even at a subclinical level [51, 83].

Although most of the significant features identified by ML are well-known risk factors for suicide [6, 7], ML demonstrate a greater predictive ability when compared with classical univariate statistics (i.e., logistic regression) and clinician assessment of risk factors [8, 9]. In particular, ML attained higher accuracies as compared to logistic regression [46, 49, 57, 61, 63, 67, 69, 87, 105]. These results suggest that advanced methods may inform the clinical decision-making processes in a more precise manner, likely overcoming the poor predictive value provided by classical statistics and expert assessment of the same risk factors [8, 9]. Interestingly, when present, CNN seemed to perform better than other ML algorithms, including SVM and RF. This might indicate the possibility of using deep learning to better stratify suicide risk, at the cost of a slight loss of interpretability.

Lastly, only a few studies employed biological features, such as genes, SNPs, epigenetic loci [42, 43, 98, 106], and neuroimaging measures [47, 49, 53, 68, 69, 79, 81] to predict suicide. Surprisingly, just a single study [53] combined brain imaging with clinical data to predict suicidal behaviors. As one of the major strengths of ML is the possibility to combine data obtained through different modalities (e.g., genetics, brain imaging, clinical features) to increase prediction accuracy, this approach should be exploited in future suicide research, since it is already occurring in other field of medicine [14].

### Limitations and future challenges

A number of limitations should be highlighted. Methods varied widely across studies in terms of ML approach, sample selection, features employed, and preprocessing pipeline. Moreover, distinct investigations focused on a variety of different outcomes, from lifetime attempts to death by suicide, from cross-sectional to longitudinal evaluations. Such differences call for increased uniformity in the assessment of suicidal behaviors and in the design of ML protocols to enhance predictions of risk that may translate into clinical practice.

For instance, the decision to use either a specific and unique ML framework or different algorithms should be motivated: the testing of several approaches at once seems confusing and rather exploratory, especially in the absence of an external validation dataset. Regarding the different algorithms, it is noteworthy to mention that, from our results, it emerged that deep learning methods (such as CNN) performed better than other ML algorithms in direct comparisons. Although important from a research point of view, deep learning algorithms tend to be less interpretable (more “black boxes”), and this aspect might prove crucial in the further development of AI techniques in medicine and psychiatry. This is true, especially in the field of mental health and suicide prediction, where AI tools should assist clinicians and not introduce further complexity. For an AI to become useful in clinical practice, it should prove to be trustworthy, therefore not only valid and reliable, but also easily understandable [107]. In the last years, the concept of explainable AI (“XAI”) emerged, as a possibility to close the gap between the algorithms and the clinicians, creating a human-understandable correspondence between inputs and outputs of the black-box model either through intrinsic transparency of the model or through post-hoc techniques. Given that clinical applications are high-stakes, we require understandability from the prediction tools, or either AI tools will grow in distrust [107].

Moreover, features should be accurately selected, and their number should not be excessive (e.g., curse of dimensionality), as in some of the studies [44, 61]. Collecting such a huge amount of data could be feasible only in university centers, thus reducing the translational value of the results. This comprehensive review should also help in the choice of the right type and number of features. For example, pharmacological treatments, especially antipsychotics, were among the most important features in those studies who included them in the models. However, the pharmacological status of patients is often not reported (see Table 1), and in most cases type and dosage of different drugs are not included in the models. Based on the results of our review, it might be beneficial to include data related to pharmacological therapy in the models, since it could potentially enhance the predictive power and clinical applicability of these models. Moreover, the inclusion of pharmacological information might also help in defining protective features, not just risk factors, as suggested by studies showing that some stabilizers and antidepressants might actually reduce the risk of suicide [64]. Also, both psychiatric and physical comorbidities seem to have a predictive role in the presented models; especially, substance abuse as a comorbid disorder resulted to be highly predictive. This aspect suggests a comprehensive evaluation of the patient in order to define the clinical risk.

In addition, most of the studies addressed the prediction of suicide using a cross-sectional approach, disregarding the temporal aspects. Yet, time may represent a crucial feature for predictive models of suicide [17]. In this regard, defining in advance one or more prediction windows after the assessment is fundamental, as the prediction of short-term suicide risk may rely on different features as compared with long-term risk. Similarly, the temporal characteristics of a feature with respect to the assessment point might impact differentially the accuracy of prediction. For instance, suicide attempts in the year prior to the assessment, but not those that occurred several years before, may be a stronger predictor for new short-time suicidal behaviors.

Finally, despite the high heterogeneity, most of the studies (>80%) obtained a good accuracy, namely 70% or higher. However, many studies did not report additional key metrics (e.g., PPV, F1-score) that are paramount to interpret the actual usefulness of prediction models. Moreover, only few studies tested their prediction on external validation samples; therefore, caution is needed when interpreting these findings, since it is possible that they suffer from overfitting.

Finally, it is evident the importance of further studies also examining the role of neurocognitive variables, dimensions of social support, loneliness, extent and type of medical comorbidity and associated disability, the type of pharmacological interventions used in the context of specific diagnoses as well as the presence of psychotherapies and their combination with medications on suicidal risk. Similarly, a call for a more consistent use of ML is of paramount importance. CNN, RF, and SVM proved to perform better against other algorithms, but these results should be further tested in the future.

## Conclusions

The results that emerged from the reviewed studies lead to the conclusion that ML approaches attain greater accuracies in predicting suicidal behaviors across a variety of psychiatric disorders as compared to classical analysis methods. From the reviewed ML studies, well-known risk factors for suicide emerged as relevant predictors, along with new subtle aspects, such as physical and psychiatric comorbidities, presence of psychotic symptoms, and subclinical lab tests, that should be further analyzed and confirmed in future studies. However, additional work is needed to improve the predictive strength of ML algorithms, resolve the systemic lack of external validation, and finally make them become of use in clinical psychiatry. To do so, ML should integrate genetics, neurobiological, brain imaging, psychometric and clinical data to achieve better predictions. Then, algorithms should be presented in an intuitive way for both psychiatrists and patients to foster their adoption and easiness of use in the clinical setting. Although some attempts have been made, to date, ML approaches are not routinely part of clinical practice in psychiatry. We believe ML development should aim to gain the trust of clinicians, by proving to be valid, reliable, and understandable, to be realistically included in decision processes. Our review proved they can be valid in the context of suicide risk stratification; future studies should demonstrate that ML tools are reliable and, even more importantly, easy to understand by clinicians. Multifactorial disorders require multifaceted approaches, and ML could really help in this aspect; however, AI tools should not introduce further complexity in the decision processes, and therefore explainable AI will be a crucial point in further clinical development of predictive tools.

### Supplementary information

Supplementary Materials

## Data Availability

All data will be made available upon request.
