# Supplementary material for: Machine learning and the prediction of suicide in psychiatric populations: a systematic review
Source: Transl Psychiatry. 2024 Mar 9;14:140. doi: 10.1038/s41398-024-02852-9 (PMC10925059; doi:10.1038/s41398-024-02852-9)
Supplement: Supplementary file 1 — Supplementary Materials [file 41398_2024_2852_MOESM1_ESM.docx]

**Supplementary Materials**

**Supplementary Methods**

Quality assessment

Quality rating of the paper included in the review was assessed following two different scores.

First, a modified version of the score proposed by Hinterwimmer and colleagues (2022) was used to assess the quality of the manuscripts. The max total score is 100. A score of 100 indicates that the study largely avoids chance, bias and other confounding factors. Further, score can be defined as excellent (85–100 points), good (70–84 points), fair (50–69 points), and poor (< 50 points). The questions presented are reported in Supplementary Table 1.

Moreover, risk of bias assessment was performed using Transparent Reporting of a Multivariable Prediction Model for Individual Prognosis or Diagnosis (TRIPOD) guidelines (Collins et al., 2015). The TRIOPD checklist contains 22 primary features as well as multiple subitems, resulting in a total of 37 features. A TRIPOD score was created using 1 possible point for each subitem, as already proposed by Subramanian et al., 2021. Adherence to a subitem was given 1 point, while non-adherence was scored as 0 points. Features not assessed in an article due to the nature of the study were deemed as not applicable and excluded from analysis. The primary features are title (1 point), abstract (1 point), introduction - background and objectives (2 points), methods - source of data (2 points), methods - participants (3 points), methods - outcome (2 points), methods - predictors (2 points), methods - sample data (1 point), methods - missing data (1 point), methods - statistical analysis (5 points), methods - risk groups (1 point), methods - development and validation (1 point), results - participants (3 points), results - model development (2 points), results - model specification (2 points), results - model performance (1 point), results - model updating (1 point), discussion - limitations (1 point), discussion - interpretation (2 points), discussion - implications (1 point), supplementary information (1 point) and funding (1 point). The individual TRIPOD ratio was calculated for each article as the ratio of the TRIPOD score to the maximum possible points calculated from the included features.

Of note, leave-one out Cross-Validation was considered a sub-standard method and therefore was cause of reduced scores in the quality assessment (Sanfelici et al., 2020).

**Supplementary Table 1**. Quality check of the reviewed studies using a modified version of the scale developed by Hinterwimmer et al. (2022).

| **Part A—only one score to be given for each of the seven sections** | | |
| --- | --- | --- |
| *1. Study size—number of patients (N)* | |  |
| N > 500 | 10 |  |
| N 100–500 | 7 |  |
| N 20–100 | 4 |  |
| N < 20 or not stated | 0 |  |
|  |  |  |
| *2. Mean follow-up* | |  |
| > = 1 years | 10 |  |
| 1–12 months | 5 |  |
| < 1 months, not stated, or unclear | 0 |  |
|  |  |  |
| *3. Type of sample* | |  |
| Prospective cohort study | 15 |  |
| Retrospective cohort study | 10 |  |
| Experimental data set | 5 |  |
|  |  |  |
| *4. Type of study* | |  |
| Multiple-outcome variables | 10 |  |
| Single-outcome variables | 5 |  |
|  |  |  |
| *5. Number of input variables* | |  |
| > 50 | 5 |  |
| 10-50 | 3 |  |
| <10 | 0 |  |
|  |  |  |
| 6*. Description of ML-approach* | |  |
|  |  |  |
| Technique stated with necessary details to repeat | 10 |  |
| Technique named without elaboration | 5 |  |
| Not stated or unclear | 0 |  |
|  |  |  |
| *7. Fine-tuning of ML-model* | |  |
| Yes (adequate CV, permutations, features engineering) | 5 |  |
| No | 0 |  |
|  |  |  |
| **Part B—scores may be given for each option in each of the three sections if applicable** | | |
| *1. Metrics* | |  |
| Suitable metrics | 5 |  |
| More than one metric stated | 5 |  |
| External dataset for final evaluation | 5 |  |
|  |  |  |
| *2. Data screening* | |  |
| Data processing elaborated and stated | 5 |  |
| Data source stated | 5 |  |
|  |  |  |
| *3. Mathematical and medical discussion* | |  |
| Metrics stated and elaborated in medical context | 5 |  |
| Metrics statistically elaborated | 5 |  |

**Supplementary Table 2.** TRIPOD Checklist.

| **Section/Topic** | **Item** | **Checklist Item** | **Page** |
| --- | --- | --- | --- |
| **Title and abstract** | | | |
| Title | 1 | Identify the study as developing and/or validating a multivariable prediction model, the target population, and the outcome to be predicted. |  |
| Abstract | 2 | Provide a summary of objectives, study design, setting, participants, sample size, predictors, outcome, statistical analysis, results, and conclusions. |  |
| **Introduction** | | | |
| Background and objectives | 3a | Explain the medical context (including whether diagnostic or prognostic) and rationale for developing or validating the multivariable prediction model, including references to existing models. |  |
|  | 3b | Specify the objectives, including whether the study describes the development or validation of the model or both. |  |
| **Methods** | | | |
| Source of data | 4a | Describe the study design or source of data (e.g., randomized trial, cohort, or registry data), separately for the development and validation data sets, if applicable. |  |
|  | 4b | Specify the key study dates, including start of accrual; end of accrual; and, if applicable, end of follow-up. |  |
| Participants | 5a | Specify key elements of the study setting (e.g., primary care, secondary care, general population) including number and location of centres. |  |
|  | 5b | Describe eligibility criteria for participants. |  |
|  | 5c | Give details of treatments received, if relevant. |  |
| Outcome | 6a | Clearly define the outcome that is predicted by the prediction model, including how and when assessed. |  |
|  | 6b | Report any actions to blind assessment of the outcome to be predicted. |  |
| Predictors | 7a | Clearly define all predictors used in developing or validating the multivariable prediction model, including how and when they were measured. |  |
|  | 7b | Report any actions to blind assessment of predictors for the outcome and other predictors. |  |
| Sample size | 8 | Explain how the study size was arrived at. |  |
| Missing data | 9 | Describe how missing data were handled (e.g., complete-case analysis, single imputation, multiple imputation) with details of any imputation method. |  |
| Statistical analysis methods | 10a | Describe how predictors were handled in the analyses. |  |
|  | 10b | Specify type of model, all model-building procedures (including any predictor selection), and method for internal validation. |  |
|  | 10d | Specify all measures used to assess model performance and, if relevant, to compare multiple models. |  |
| Risk groups | 11 | Provide details on how risk groups were created, if done. |  |
| **Results** | | | |
| Participants | 13a | Describe the flow of participants through the study, including the number of participants with and without the outcome and, if applicable, a summary of the follow-up time. A diagram may be helpful. |  |
|  | 13b | Describe the characteristics of the participants (basic demographics, clinical features, available predictors), including the number of participants with missing data for predictors and outcome. |  |
| Model development | 14a | Specify the number of participants and outcome events in each analysis. |  |
|  | 14b | If done, report the unadjusted association between each candidate predictor and outcome. |  |
| Model specification | 15a | Present the full prediction model to allow predictions for individuals (i.e., all regression coefficients, and model intercept or baseline survival at a given time point). |  |
|  | 15b | Explain how to use the prediction model. |  |
| Model performance | 16 | Report performance measures (with CIs) for the prediction model. |  |
| **Discussion** | | | |
| Limitations | 18 | Discuss any limitations of the study (such as nonrepresentative sample, few events per predictor, missing data). |  |
| Interpretation | 19b | Give an overall interpretation of the results, considering objectives, limitations, and results from similar studies, and other relevant evidence. |  |
| Implications | 20 | Discuss the potential clinical use of the model and implications for future research. |  |
| **Other information** | | | |
| Supplementary information | 21 | Provide information about the availability of supplementary resources, such as study protocol, Web calculator, and data sets. |  |
| Funding | 22 | Give the source of funding and the role of the funders for the present study. |  |

**Supplementary Table 3.** Quality rating and bias assessment of the studies included in the systematic review.

| **Author and year** | **Quality score** | **TRIPOD score** | **TRIPOD ratio** |
| --- | --- | --- | --- |
| Wang et al., 2022 | 70 | 19 | 0.95 |
| Chen et al., 2022 | 72 | 17 | 0.85 |
| Chen et al., 2022 | 67 | 17 | 0.85 |
| Zhong et al., 2022 | 69 | 17 | 0.85 |
| Xu et al., 2022 | 73 | 18 | 0.9 |
| Zheng et al., 2022 | 70 | 18 | 0.9 |
| Shin et al., 2022 | 69 | 15 | 0.75 |
| Miranda et al., 2022 | 70 | 14 | 0.7 |
| Zelkowitz et al., 2022 | 80 | 18 | 0.9 |
| Tubío-Fungueiriño et al., 2022 | 75 | 17 | 0.85 |
| Yang et al., 2022 | 67 | 15 | 0.75 |
| Nock et al., 2022 | 90 | 19 | 0.95 |
| Shao et al., 2021 | 72 | 17 | 0.85 |
| Kim et al., 2021 | 72 | 17 | 0.85 |
| Ji et al., 2021 | 67 | 16 | 0.8 |
| Li et al., 2021 | 80 | 17.5 | 0.87 |
| Jiang et al., 2021 | 75 | 18 | 0.9 |
| McMullen et al., 2021 | 78 | 17 | 0.85 |
| Coley et al., 2021 | 90 | 17 | 0.85 |
| Chen et al., 2021 | 77 | 17 | 0.85 |
| Liu et al., 2021 | 77 | 17 | 0.85 |
| Nordin et al., 2021 | 67 | 15 | 0.75 |
| Nestsiarovich et al., 2021 | 75 | 15.5 | 0.77 |
| Jiang et al., 2021 | 85 | 17 | 0.85 |
| Adams et al., 2021 | 70 | 15.5 | 0.77 |
| Cusick et al., 2021 | 75 | 16 | 0.8 |
| Bohaterewicz et al., 2021 | 69 | 17 | 0.85 |
| Machado et al., 2021 | 93 | 18 | 0.9 |
| Edgcomb et al., 2021 | 85 | 18 | 0.9 |
| Iorfino et al., 2020 | 78 | 17 | 0.85 |
| Zhu et al., 2020 | 72 | 15 | 0.75 |
| Edgcomb et al., 2021 | 85 | 18 | 0.9 |
| Hong et al., 2021 | 69 | 16 | 0.8 |
| Parghi et al., 2021 | 78 | 17 | 0.85 |
| Chen et al., 2020 | 90 | 18 | 0.9 |
| Dai et al., 2020 | 64 | 15 | 0.75 |
| Fan et al., 2020 | 70 | 16 | 0.8 |
| Obeid et al., 2020 | 75 | 16 | 0.8 |
| Agne et al., 2020 | 75 | 17 | 0.85 |
| Haines-Delmont et al., 2020 | 69 | 15 | 0.75 |
| Kessler et al., 2020 | 95 | 18 | 0.9 |
| Roglio et al., 2020 | 75 | 17 | 0.85 |
| Senior et al., 2020 | 69 | 15 | 0.75 |
| Ge et al., 2020 | 63 | 13 | 0.65 |
| Weng et al., 2020 | 72 | 17 | 0.85 |
| Tasmin et al., 2020 | 55 | 12 | 0.6 |
| Kumar et al., 2020 | 75 | 16 | 0.8 |
| Bhak et al., 2020 | 67 | 15 | 0.75 |
| Xu et al., 2020 | 83 | 17 | 0.85 |
| Peis et al., 2019 | 70 | 13 | 0.65 |
| Gosnell et al., 2019 | 70 | 15 | 0.75 |
| Carson et al., 2019 | 69 | 17 | 0.85 |
| Fernandes et al., 2018 | 65 | 13 | 0.65 |
| Jordan et al., 2018 | 80 | 17 | 0.85 |
| Oh et al., 2017 | 78 | 17 | 0.85 |
| Hettige et al., 2017 | 70 | 18 | 0.9 |
| Setoyama et al., 2016 | 69 | 15 | 0.75 |
| Pestian et al., 2017 | 62 | 13 | 0.65 |
| Barros et al., 2017 | 80 | 16 | 0.8 |
| Cook et al., 2016 | 70 | 15 | 0.75 |
| Kessler et al., 2017 | 75 | 16 | 0.8 |
| Walsh et al., 2017 | 75 | 17 | 0.85 |
| Morales et al., 2017 | 75 | 16 | 0.8 |
| Passos et al., 2016 | 65 | 17 | 0.85 |
| Levey et al., 2016 | 84 | 16 | 0.8 |
| Niculescu et al., 2015 | 84 | 16 | 0.8 |
| Kessler et al., 2015 | 85 | 17 | 0.85 |
| Tran et al., 2015 | 75 | 14 | 0.7 |
| Poulin et al., 2014 | 67 | 15 | 0.75 |
| Delgado-Gomez et al., 2011 | 65 | 12 | 0.6 |
| Lopez-Castroman et al., 2011 | 68 | 16 | 0.8 |
| Baca-Garcia et al., 2010 | 75 | 13 | 0.65 |
| Ilgen et al., 2009 | 50 | 11 | 0.55 |
| Mann et al., 2008 | 65 | 15 | 0.75 |
| Baca-Garcia et al., 2007 | 65 | 14 | 0.7 |
| Tiet et al., 2006 | 70 | 15 | 0.75 |
| Modai et al., 2004 | 63 | 14 | 0.7 |
| Modai et al., 2004 | 63 | 13 | 0.65 |
| Modai et al., 2002 | 57 | 10 | 0.5 |
| Modai et al., 1999 | 60 | 10 | 0.5 |
| Modai et al., 1998 | 57 | 10 | 0.5 |

**Supplementary table 4**: the table reports the winning algorithm in those studies that evaluated more than one algorithm, and the variables employed

| **Author and year** | **Population** | **Features** | **Winning Algorithm** | **Other algorithms** |
| --- | --- | --- | --- | --- |
| Wang et al., 2022 | Mood disorders | Sociodemographic features and Text mining | CNN | LR  SVM |
| Zelkowitz et al., 2022 | Mixed diagnoses | Demographic and clinical variables | RF | CART |
| Kim et al., 2021 | Adolescents with mixed diagnoses | Demographic and clinical variables | RF and SVM | LR,  ANN,  XGB |
| Ji et al., 2022 | MDD | clinical variables from clinical questionnaires | SVM | AdaBoost,  NB |
| McMullen et al., 2021 | Psychiatric patients | clinical features from the SCI and suicidal ideation | gradient boosted trees | LR,  RF |
| Nordin et al., 2021 | MDD | Demographic and clinical variables | SVM | LR,  decision tree,  naïve Bayes,  k-nearest neighbors,  RF,  bagging and voting |
| Adams et al., 2021 | SUD | Demographic and clinical variables | RF | Classification tree |
| Cusick et al., 2021 | MDD | NLP | CNN | SVM,  Naive Bayes classifier |
| Bohaterewicz et al., 2021 | SKZ | rsMRI | LASSO | GB,  LASSO,  LR,  RF,  SVM |
| Machado et al., 2021 | MDD | stressful life events, and sociodemographic variables | Elastic net regularization | RF,  ANN |
| Iorfino et al., 2020 | Mixed diagnoses | demographic and clinical variables | BART | AUCRF,  Boruta,  Lasso regression,  Elastic-net regression,  LR |
| Parghi et al., 2021 | Mixed diagnoses | features from the Suicide Crisis Inventory | gradient boosting | LR,  RF |
| Fan et al., 2020 | Both PTSD and BD | demographic and clinical variables | RF | LR,  decision tree,  K-nearest neighbors,  Naïve Bayes,  SVM |
| Obeid et al., 2020 | Intentional self harm | Text from clinical notes | CNN | Naïve Bayes,  decision tree,  RF,  SVM,  multilayer perceptron |
| Haines-Delmont et al., 2020 | Mixed diagnoses | features from sleep data, journal entries, data usage, mood, and app activity statistics | K-nearest neighbors | RF,  LR,  SVM |
| Weng et al., 2020 | MDD | features extracted from diffusion brain imaging | CNN | XGB,  LR |
| Tasmin et al., 2020 | SKZ | Clinical variables | classification tree | LR,  RF |
| Kumar et al., 2020 | Mixed diagnoses | Demographic and clinical variables | Tree-based XGboost | LR,  RF,  decision tree,  linear SVC |
| Hettige et al., 2017 | SKZ | Demographic and clinical variables | elastic net | LASSO,  RF,  SVM |
| Barros et al., 2017 | Mixed diagnoses | Demographic and clinical variables | SVM and RF | CART,  k-nearest neighbor,  AdaBoost,  NN multilayer perceptron, |
| Kessler et al., 2017 | Mixed diagnoses | Demographic and clinical variables | elastic net | naive Bayes,  RF,  SVR |
| Passos et al., 2016 | MDD and BD | Demographic and clinical variables | RVM | LASSO,  SVM |

**Supplementary table 5**: most relevant clinical variables in different studies assessing mood disorders and mixed diagnoses

| **Clinical features** | **Study** |
| --- | --- |
| **Mood disorders** | |
| History of suicide | Nordin et al., 2021  Machado et al., 2021  Edgcomb et al., 2021 |
| Family history of suicide | Wang et al., 2022 |
| Substance Use disorders | Wang et al., 2022  Fan et al., 2020  Passos et al., 2016  Ilgen et al., 2009 |
| Comorbidities | Nordin et al., 2021  Machado et al., 2021  Fan et al., 2020  Passos et al., 2016 |
| Social problems (alone, widowed etc) | Wang et al., 2022 |
| Clinical severity | Zheng et al., 2022  Li et al., 2021  Nordin et al., 2021  Ge et al., 2020 |
| Presence of Antipsychotics | Nestsiarovich et al., 2021  Fan et al., 2020 |
| fT4 | Li et al., 2021  Ge et al., 2020 |
| Previous hospital admissions | Machado et al., 2021  Passos et al., 2016  Ilgen et al., 2009 |
| **Mixed diagnoses** | |
| Substance Use disorders | Zelkowitz et al., 2022  Jiang et al., 2021  Adams et al., 2021  Kumar et al., 2020  Lopez-Castroman et al., 2011 |
| History of suicide | Nock et al., 2022  Iorfino et al., 2020  Chen et al., 2020  Kessler et al., 2020  Kessler et al., 2015 |
| Comorbidities | Zelkowitz et al., 2022  Adams et al., 2021  Chen et al., 2020  Kessler et al., 2020  Kumar et al., 2020  Mann et al., 2008 |
| Clinical severity | Kim et al., 2021  Jiang et al., 2021  Chen et al., 2020  Kessler et al., 2020  Walsh et al., 2017 |
| Social problems (alone, widowed etc) | Kim et al., 2021  Jiang et al., 2021  Iorfino et al., 2020 |
| Presence of Antipsychotics | Iorfino et al., 2020  Chen et al., 2020  Walsh et al., 2017  Kessler et al., 2015 |

**References**

Hinterwimmer F, Lazic I, Suren C, Hirschmann MT, Pohlig F, Rueckert D, Burgkart R, von Eisenhart-Rothe R. Machine learning in knee arthroplasty: specific data are key-a systematic review. Knee Surg Sports Traumatol Arthrosc. 2022 Jan 10.

Collins GS, Reitsma JB, Altman DG, Moons KG. Transparent Reporting of a multivariable prediction model for Individual Prognosis Or Diagnosis (TRIPOD): the TRIPOD Statement. Br J Surg. 2015 Feb;102(3):148-58.

Sanfelici R, Dwyer DB, Antonucci LA, Koutsouleris N. Individualized Diagnostic and Prognostic Models for Patients With Psychosis Risk Syndromes: A Meta-analytic View on the State of the Art. Biol Psychiatry. 2020 Aug 15;88(4):349-360.

Subramanian H, Dey R, Brim WR, Tillmanns N, Cassinelli Petersen G, Brackett A, Mahajan A, Johnson M, Malhotra A, Aboian M. Trends in Development of Novel Machine Learning Methods for the Identification of Gliomas in Datasets That Include Non-Glioma Images: A Systematic Review. Front Oncol. 2021 Dec 23;11:788819.
